# Supplementary material for: Gene Therapy for Glaucoma by Ciliary Body Aquaporin 1 Disruption Using CRISPR-Cas9
Source: Mol Ther. 2020 Jan 10;28(3):820–9. doi: 10.1016/j.ymthe.2019.12.012 (PMC7054720; doi:10.1016/j.ymthe.2019.12.012)
Supplement: Document S1. Figures S1–S4, Tables S1–S3, and Supplemental Materials and Methods [file mmc1.pdf]

YMTHE, Volume *28*

Supplemental Information

Gene Therapy for Glaucoma by Ciliary Body Aquaporin 1 Disruption Using CRISPR-Cas9

Jiahui Wu, Oliver H. Bell, David A. Copland, Alison Young, John R. Pooley, Ryea Maswood, Rachel S. Evans, Peng Tee Khaw, Robin R. Ali, Andrew D. Dick, and Colin J. Chu

Supplemental Figures:

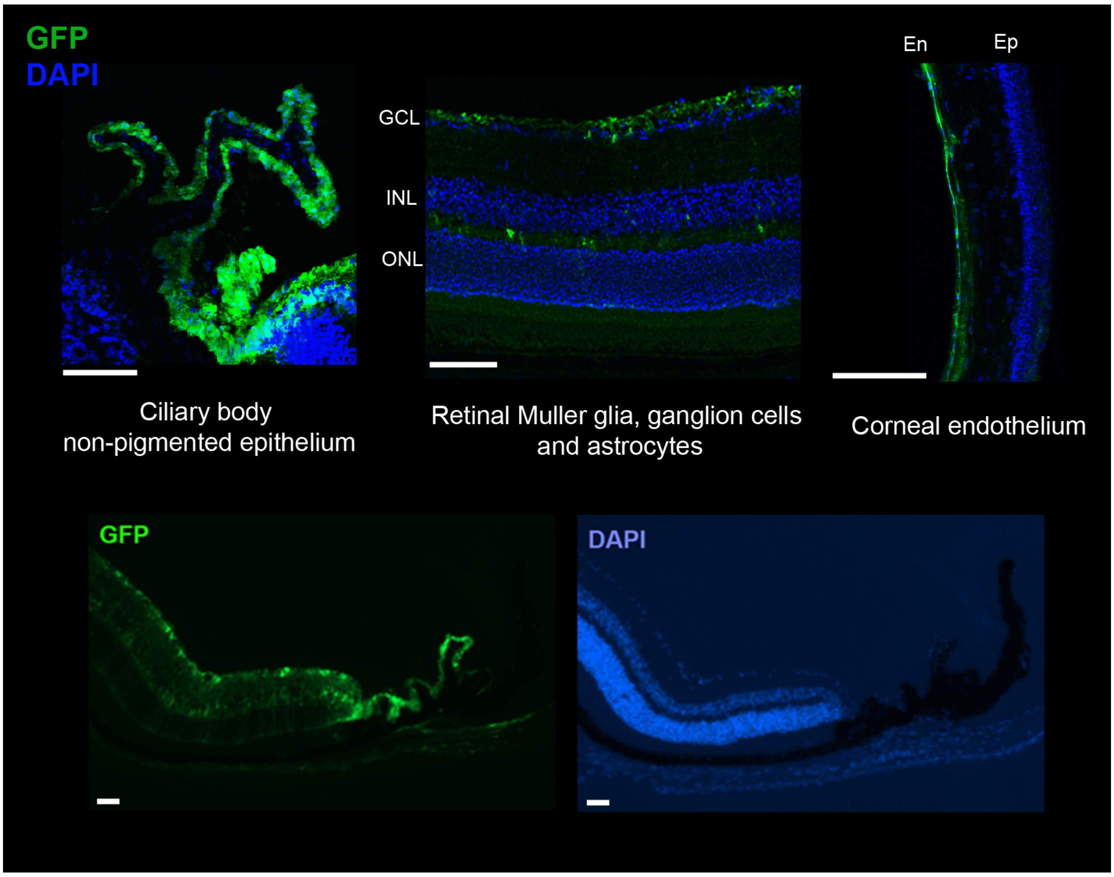

Figure S1. ShH10 produces transduction of ciliary body non-pigmented epithelium, retinal astrocytes, Müller glia, ganglion cells and corneal endothelium. Representative confocal images of *C57BL/6J* mouse eyes four weeks following intravitreal injection of 2×10^{10} genome copies of ShH10-CMV-eGFP. GCL: ganglion cell layer, INL: inner nuclear layer, ONL: outer nuclear layer, En: corneal endothelium, Ep: corneal epithelium. Scale bars: 100 µm.

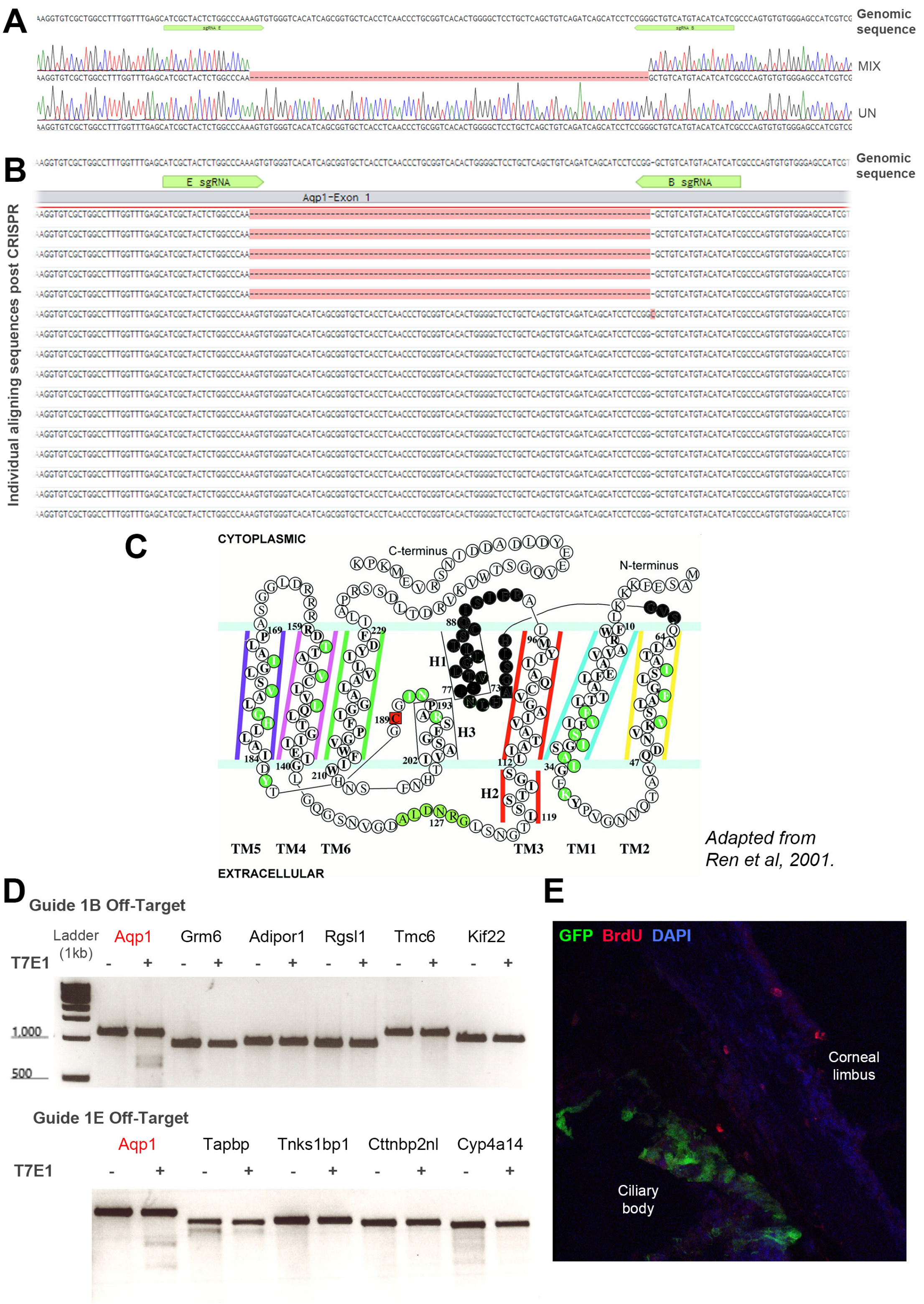

Figure S2. Representative sequencing chromatograms (**A**) from ShH10-CMV-SaCas9-sgRNA B and E (MIX) and un-infected control (UN) B6-RPE07 cells after 72 hours. Graphical depiction of on-target genomic DNA alterations (**B**) of further 16 accurately sequenced clones, where the predominant gene edit observed (31.2%) was complete excision of the 98bp intervening region between the cleavage points of the two sgRNA guides. This excised DNA region corresponds to critical central amino acid residues

shaded in black on the polypeptide sequence (**C**) adapted from *Ren et al.*[1] T7 endonuclease 1 assay for the top five bioinformatics predicted off-target coding genes (**D**) for sgRNA B and E do not demonstrate genomic cleavage.[2] Tested 72 hours following plasmid transfection into B6-RPE07 cells. Each shown with and without addition of T7E1 enzyme, with *Aqp1* positive control. To exclude ShH10 viral infection causing ciliary body non-pigmented epithelium cell turnover, BrdU was administered 24 hours before cryosection and staining, but 3 weeks after intravitreal injection of ShH10-CMV-eGFP. Representative image (**E**) of n=3 eyes showing expected corneal limbal transient amplifying cells as positive control. There is minimal colocalization of cells in the GFP+ ciliary epithelium with BrdU.

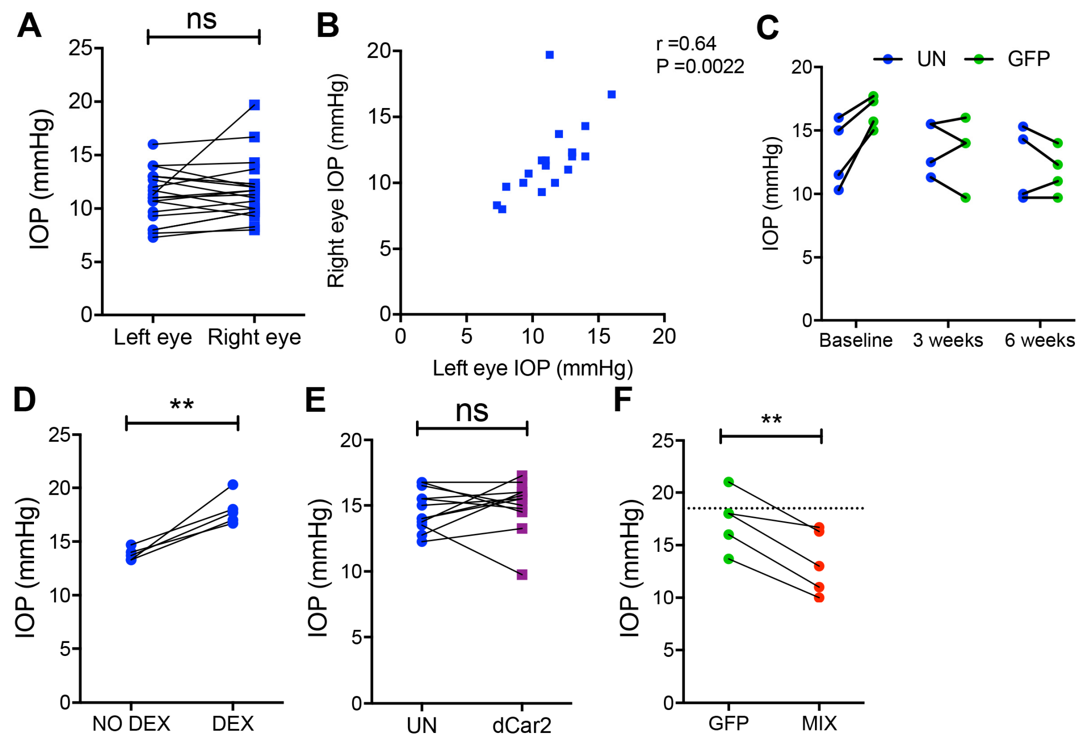

Figure S3. Additional *in vivo* data. Comparison of left and right eye intraocular pressures (IOP) prior to any intervention demonstrating no statistical difference by paired analysis (**A**, paired Student's t test, n = 19, p = 0.31) and good correlation (**B**, Pearson correlation, r = 0.64, p = 0.0022). This supports use of the contralateral eye as a control with lower inter- than intra-ocular variance (4.86 vs 5.44mmHg). IOP measurement of contralateral eyes injected with GFP vector demonstrates no statistically significant reduction from the vector across time (**C**, Repeated measures two-way ANOVA, n = 4, p = 0.52). Confirmation of the magnitude of IOP elevation in the corticosteroid (Dexamethasone) ocular hypertension model is shown as IOP after 3 weeks with contralateral eye control (**D**, paired Student's t test, n = 5, p = 0.0052). Delivery of catalytically inactive Cas9 and sgRNA targeting a different gene (*Car2*) does not reduce IOP (**E**, n = 12, paired Student's t test, n = 12, p = 0.62), implying potential immune response against intravitreal injection, AAV or Cas9 expression may not affect IOP. GFP control vector was injected contralaterally alongside the MIX vector in an independent experiment one week following microbead model induction. After three weeks, reduced IOP is still observed (**F**, paired Student's t test, n = 5, P = 0.005, dotted line is mean IOP pre-AAV injection). Mean ± SEM shown. ns = not statistically significant.

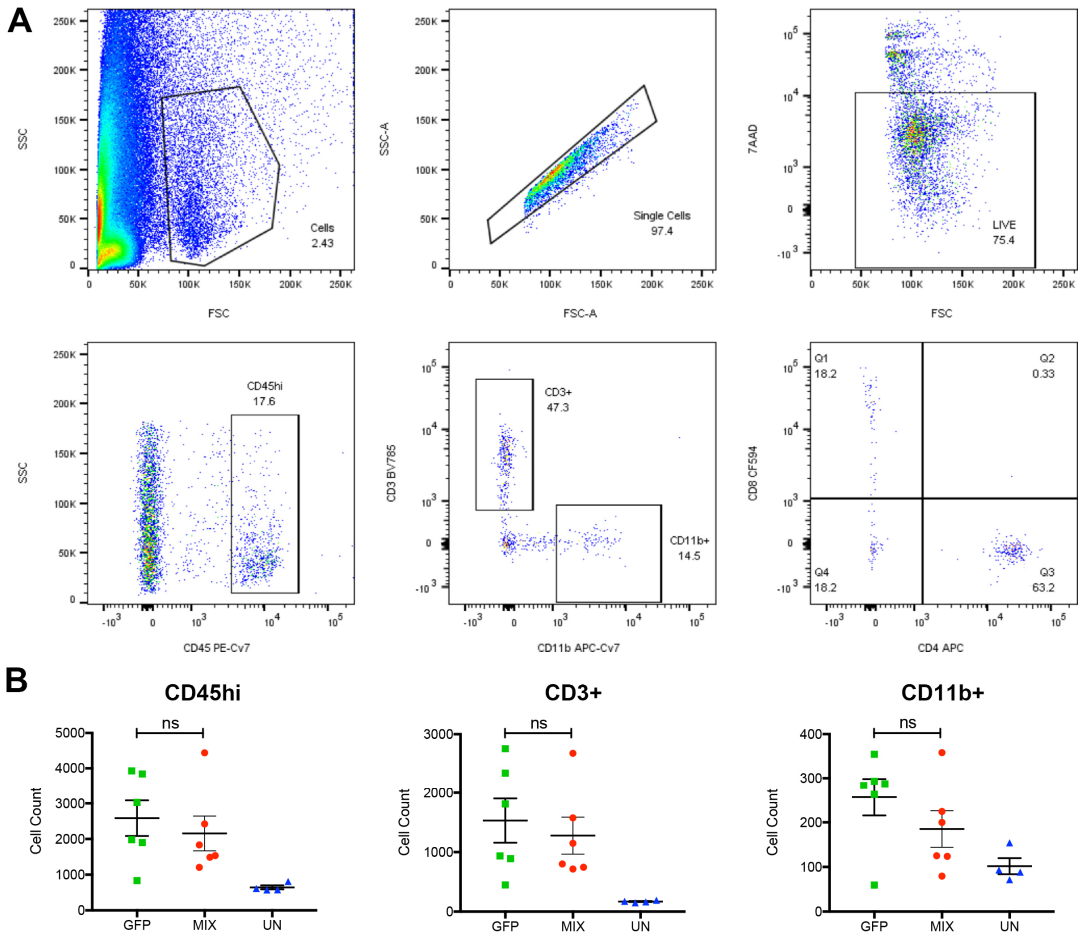

Figure S4. Ciliary body flow cytometry does not demonstrate differences in immune cell infiltration between the CRISPR-Cas9 and GFP control vectors. Whilst immune responses in the eye following intravitreal AAV injection can occur, we fail to identify differences between MIX and GFP that could confound the reduction in IOP observed. Eyes were injected with either ShH10-CMV-eGFP, un-injected (UN) or *Aqp1* targeting MIX. Three weeks later the ciliary body was extracted and analysed by flow cytometry for immune cell infiltration. **A**) Gating strategy on representative MIX eye and **B**) summary graph of absolute cell counts for total infiltrating leukocytes ($CD45^{Hi}$), lymphocytes (CD45+CD3+) or myeloid (CD45+CD11b+) populations. One-way ANOVA with Holm-Sidak's multiple comparison test between GFP and MIX, n=6 eyes per group. Mean ± SEM shown. ns = not statistically significant.

Supplemental Tables

Supplemental Table 1. CRISPR-SaCas9 sgRNA sequences

Target gene	sgRNA name	sgRNA sequence
Mouse *Aqp1 exon 1*	A	GATGTGACCCACACTTTGGGC
	B	GATGATGTACATGACAGCCCG
	C	TCTTCTGGAGGGCTGTGGTGG
	D	ACCAATGCTGATGAAGACGAA
	E	ATCGCTACTCTGGCCCAAAGT
	F	TAGGGAGGAGGTGATGCCCGA
Human *AQP1 exon 1*	H	CTGAGCATCGCCACGCTGGCG
	J	ACCGATGCTGATGAAGACAAA
	K	CCGCCGTCTGGTTGTTCCCCA

Supplemental Table 2. Primers for endpoint PCR and qPCR

Application and gene target	Forward Primer	Reverse Primer
T7 endonuclease 1 assay - Mouse *Aqp1* exon 1	CTAGAGTGCCAGCCTCTGCCCT	GGAGGAACTGCTGGCATGCACC
T7 endonuclease 1 assay - Human *AQP1* exon 1	CTGCATCCATCCAGAGGAGGTC	GACAGGCTGGGCATTTGGTTC
Endpoint PCR for SaCas9 DNA sequence	AGCAAGGCCCTGGAAGAGAAAT	TGGTGGGGATCTCTTTCTGCTG

Supplemental Table 3. T7 endonuclease 1 assay primers for predicted region of CRISPR-Cas9 off-target coding genes

sgRNA	Predicted off-target gene	Forward Primer	Reverse Primer
m*Aqp1* B	Tmc6	CTCGCACATGAGCACTGTCTTC	TGGCTCAGCAATTAAGAGGGCT
m*Aqp1* B	Kif22	GCACTTCCTTCTGCTGGCAAAA	CTACTGGCTGGAAGGAAGTGCT
m*Aqp1* B	Grm6	CTATAGGTAGCGCAGGAACCCC	TATGCACCCCTTAGGCACTCAC
m*Aqp1* B	Adipor1	CACCACTATGCTAGCTCCCTGT	GGGACAGTGAGCTGACATGTCA
m*Aqp1* B	Rgsl1	ATGGGACCTGATGTGTGTCCTG	GATGGTTTCTGCCTTGGCAGAC
m*Aqp1* E	Tapbp	ATTGTGCCCTTAGAGATCCGGG	CTCTGAGCTCCCACTTGACCTC
m*Aqp1* E	Tnks1bp1	TTCTTCCATCTGCCTCCCTGTG	TCACCACGTCCTCCTCAGTAGA
m*Aqp1* E	Cttnbp2nl	TAATGGCCACTGTGACCCAGAG	GGGTTTAACGCTGAGCTCTTGG
m*Aqp1* E	Cyp4a14	TCGCTTTAACTCTGACCAGCCA	TGGTGAGAGTGGGGTGTTGAAG

Supplemental Materials & Methods

CRISPR-Cas9 indel formation characterisation

B6-RPE07 at 70% confluency in a 24-well plate were transfected using Lipofectamine 3000 (Thermo Fisher Scientific, UK) with a 50:50 mix of pX601-AAV-CMV:NLS-SaCas9-NLS-3xHA-bGHpA;U6::BsaI-sgRNA plasmid containing sgRNA mAqp1 B or E. After 72 hours genomic DNA was isolated using DNeasy Blood and Tissue Kit (Qiagen, Germany) and the edited exon 1 *mAqp1* region amplified using PCR Master Mix (Thermo Fisher Scientific, UK) and primers 5'-CTAGAGTGCCAGCCTCTGCCCT-3' and 5'- GGAGGAACTGCTGGCATGCACC-3'. The resulting amplicon mix was ligated into pGEM-T Easy plasmid (Promega Corporation, WI, USA) per manufacturer's instructions and transformed into DH5α competent cells (Thermo Fisher Scientific, UK). Twenty individual colonies were isolated, plasmid DNA extracted and sent for Sanger sequencing (Eurofins Genomics, Germany) using primer 5'-TGTAAAACGACGGCCAGT-3'. Alignment of resulting sequences was performed using Benchling.

BrdU cell division assay

One-hundred microlitres of 10mg/ml Bromodeoxyuridine solution (BD Biosciences, CA, USA) was injected intraperitoneally into mice 24-hours prior to termination. Eyes were enucleated and cryo-sectioned at 14µm intervals as described previously. After washing with PBS, sections were incubated with 2N HCL at 37°C for 15 minutes then processed with primary and secondary antibodies as per manufacturer's instructions (eBioscience™ BrdU Kit for IHC/ICC Immunofluorescence eFluor™ 570, Thermo Fisher Scientific, UK). Sections were imaged by confocal laser scanning microscopy on a Leica SP5 (Leica Microsystems, Germany).

Flow cytometry of Ciliary Body

Eyes were dissected in 100µl PBS with ciliary body, iris and aqueous extracted following a limbal incision, lens removal and transfer to a 1.5 mL microcentrifuge tube. Tissue was mechanically dissociated rapping the tube across an 80-well rack ten times before transfer into a 96-well 60 µm cell strainer plate (Merck Millipore, UK) which was centrifuged at 1200 rpm for 5 minutes before the cell pellet was resuspended in 0.1% BSA in PBS buffer and transferred into a 96-well V-bottom plate. Cell pellets were incubated with purified rat anti-mouse CD16/32 Fc block (1:50, 553142, [2.4G2], BD Biosciences (BD)) for 10 minutes on ice before incubation with fluorochrome-conjugated monoclonal antibodies against mouse immune cell surface markers: CD45 [1:1500, 552848, [30-F11], BD), CD3 (1:40, 100355, [145-2C11], BioLegend), CD4 (1:100, 553051, [RM4-5], BD), CD8 (1:200, 126622, [YTS156.7.7], BioLegend) and CD11b (1:200, 557657, [M1/70], BD) at 4°C for 20 minutes. Following washing cells were resuspended in 200 µl 7-aminoactinomycin D (7AAD; Thermo Fisher) and acquired using a fixed and stable flow rate for 4 minutes on a 4-laser Fortessa X20 flow cytometer (BD Cytometry Systems, UK). Compensation was performed using OneComp eBeads (01-1111-41, ThermoFisher) and eight two-fold serial dilutions of a known concentration of AccuCheck Counting Beads (PCB100, ThermoFisher) were similarly acquired to construct a standard curve to calculate absolute cell numbers. Final analysis was performed using FlowJo software (Treestar, California, USA).

Supplemental References

1. Hsu P, Scott D, Weinstein J, Ran F, Konermann S, Agarwala V, *et al* (2013). DNA targeting specificity of RNA-guided Cas9 nucleases. *Nature Biotechnology* **31**:827-32.
