# Supplementary material for: Gene Therapy for Glaucoma by Ciliary Body Aquaporin 1 Disruption Using CRISPR-Cas9
Source: Mol Ther. 2020 Jan 10;28(3):820–9. doi: 10.1016/j.ymthe.2019.12.012 (PMC7054720; doi:10.1016/j.ymthe.2019.12.012)
Supplement: Document S2. Article plus Supplemental Information [file mmc2.pdf]

Molecular Therapy
Original Article

Gene Therapy for Glaucoma by Ciliary Body Aquaporin 1 Disruption Using CRISPR-Cas9

Jiahui Wu,[1,2] Oliver H. Bell,[1] David A. Copland,[1] Alison Young,[1] John R. Pooley,[1] Ryea Maswood,[3] Rachel S. Evans,[5] Peng Tee Khaw,[3,4] Robin R. Ali,[3,4] Andrew D. Dick,[1,3,4] and Colin J. Chu[1]

[1]Translational Health Sciences, University of Bristol, Bristol BS8 1TD, UK; [2]Department of Ophthalmology, Shanghai General Hospital, Shanghai Jiao Tong University School of Medicine, Shanghai 200080, China; [3]UCL Institute of Ophthalmology, 11-43 Bath Street, London EC1V 9EL, UK; [4]NIHR Biomedical Research Centre for Ophthalmology at Moorfields Eye Hospital and UCL Institute of Ophthalmology, London EC1V 2PD, UK; [5]John van Geest Centre for Brain Repair, Department of Clinical Neurosciences, University of Cambridge, Cambridge CB2 0PY, UK

Glaucoma is a common cause of blindness, yet current therapeutic options are imperfect. Clinical trials have invariably shown that reduction in intraocular pressure (IOP) regardless of disease subtype prevents visual loss. Reducing ciliary body aqueous humor production can lower IOP, and the adeno-associated virus ShH10 serotype was identified as able to transduce mouse ciliary body epithelium following intravitreal injection. Using ShH10 to deliver a single vector CRISPR-Cas9 system disrupting Aquaporin 1 resulted in reduced IOP in treated eyes (10.4 ± 2.4 mmHg) compared with control (13.2 ± 2.0 mmHg) or non-injected eyes (13.1 ± 2.8 mmHg; $p < 0.001$; $n = 12$). Editing in the aquaporin 1 gene could be detected in ciliary body, and no off-target increases in corneal or retinal thickness were identified. In experimental mouse models of corticosteroid and microbead-induced ocular hypertension, IOP could be reduced to prevent ganglion cell loss (32 ± 4 /mm^2) compared with untreated eyes (25 ± 5/mm^2; $p < 0.01$). ShH10 could transduce human ciliary body from post-mortem donor eyes in *ex vivo* culture with indel formation detectable in the Aquaporin 1 locus. Clinical translation of this approach to patients with glaucoma may permit long-term reduction of IOP following a single injection.

INTRODUCTION

Glaucoma is the commonest cause of irreversible blindness worldwide, affecting more than 64 million people.[1] No cure exists, but multiple clinical trials have shown that regardless of the initiating pathology or disease subtype, sustained reduction in intraocular pressure (IOP) can prevent retinal ganglion cell death, optic nerve damage, and subsequent visual loss.[2–4] Given the requirement for ongoing IOP control following diagnosis, present treatment options remain imperfect, and management is challenging even in developed healthcare systems.[5] IOP-reducing drops are used initially but have frequent side effects, are only moderately efficacious, and concordance is typically poor because daily administration is required.[6,7] Glaucoma surgery (e.g., trabeculectomy) is effective but requires access to highly trained surgeons, needs intensive monitoring, risks potentially catastrophic complications, and often fails over time.[8–10]

IOP arises as a balance between the formation of aqueous humor by the ciliary body and outflow via the uveoscleral route or trabecular meshwork.[11] Most interventions focus on increasing outflow; however, ciliary body destructive procedures (e.g., cyclodiode laser) universally and markedly reduce IOP even in severe end-stage glaucoma. However, the resulting ciliary body necrosis can lead to inflammation, loss of neurotrophic factors, and subsequent phthisis bulbi that has limited the wider use of this approach.[12–14]

Gene editing to provide a single but permanent therapeutic alteration is an appealing approach given glaucoma is a chronic disease that requires lifelong intervention. Progress to date has remained at preclinical stages, with methods focused on either modulating the trabecular meshwork to increase outflow[15,16] or providing neuroprotection to ganglion cells.[17,18] A prior study has used CRISPR-Cas9 to correct one mutation in the myocilin (*MYOC*) gene associated with the development of glaucoma.[19] However, glaucoma is rarely monogenic in origin, and many genes have been associated with increased risk, each of which can possess multiple pathogenic mutations.[20,21] Nearly a hundred mutations have been identified in myocilin alone, making such specific gene-editing approaches less translationally viable.

In this study, we demonstrate a pragmatic gene therapy approach that reduces IOP by selectively disrupting aqueous humor production in the ciliary body following a single intravitreal injection. Specific gene editing is achieved using the smaller *S. aureus*-derived CRISPR-Cas9 platform, because it is capable of being delivered within a single recombinant adeno-associated virus (AAV), the vector that is now the gold standard and US Food and Drug Administration (FDA) licensed for ocular gene therapy.[22,23] By targeting a gene critical to a conserved physiological process rather than correcting one specific mutation, universal application without a prohibitive personalized approach is tractable. IOP reduction is achieved by disrupting Aquaporin 1 (*Aqp1*) in

Received 10 July 2019; accepted 30 December 2019;
https://doi.org/10.1016/j.ymthe.2019.12.012.

Correspondence: Colin J. Chu, Translational Health Sciences, University of Bristol, Bristol BS8 1TD, UK.
E-mail: colin.chu@bristol.ac.uk

 © 2020 The Author(s).
This is an open access article under the CC BY license (http://creativecommons.org/licenses/by/4.0/).

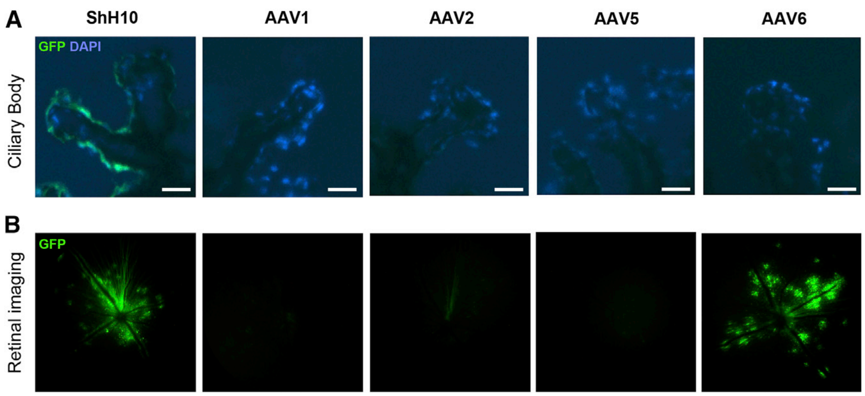

Figure 1. Adeno-Associated Virus ShH10 Serotype Efficiently Transduces Ciliary Body Epithelium following Intravitreal Injection

A total of 2 × 10^{10} genome copies of different AAV serotypes encoding GFP driven by the CMV promoter were injected into the vitreous cavity of one eye of each mouse. (A and B) Four weeks later, (A) ciliary body expression was compared using immunofluorescent sections, and (B) retinal transduction was examined by *in vivo* fundal fluorescence imaging. Only the ShH10 serotype demonstrated clear ciliary body GFP expression. Representative images from n = 8, two independent experiments. Scale bars, 25 μm.

the ciliary body. Aquaporins are a family of water-transporting transmembrane proteins that are widely expressed throughout the human body,[24,25] and transgenic mice deficient in *Aqp1* have been shown to have lower IOP as a result of reduced inflow and aqueous humor formation without adverse effect.[26]

RESULTS

ShH10 Virus Transduces Mouse Ciliary Body Epithelium following Intravitreal Injection

Five serotypes of AAV encoding GFP under the control of the constitutive cytomegalovirus (CMV) promoter were injected into mouse eyes via the intravitreal route. The ShH10 serotype was the only virus to demonstrate strong GFP expression in ciliary body non-pigmented epithelium by 4 weeks (Figure 1A). Retinal *in vivo* imaging revealed additional GFP expression around the optic disc and retinal vessels using ShH10 (Figure 1B). This was found to predominantly arise from Müller glia, ganglion cells, and astrocytes by morphology and anatomical location. Weak GFP signal was observed in the corneal endothelium (Figure S1).

Combining Two CRISPR-Cas9 Short Guide RNAs Leads to Efficient Aquaporin 1 Disruption

Relative *Aqp1* protein and transcript levels were assessed across different tissues of the mouse eye (Figures 2A–2C) and identified in the ciliary body, cornea, and retinal pigment epithelium (RPE). No AQP1 protein was detected in the retina, implying any off-target retinal transduction would not be detrimental. Six *Staphylococcus aureus*-derived Cas9 (SaCas9)-compatible short guide RNA (sgRNA) sequences within exon 1 of *Aqp1* were identified (Figure 2D). These were cloned into a single plasmid also encoding SaCas9, transiently transfected into the mouse B6-RPE07 cell line, and tested for indel formation efficiency by T7 endonuclease 1 assay (Figure 2E). Two sgRNAs (named B and E) were selected as a compromise between efficacy and spacing across exon 1 and were each produced as single AAV vectors using the ShH10 serotype capsid. More efficient gene disruption was observed when targeting with two sgRNAs compared with a single sgRNA approach, a finding also noted in other studies.[27,28] Therefore, a 1:1 ratio of sgRNAs B and E was employed and henceforth denoted as "MIX." Culturing B6-RPE07 cells with these viruses *in vitro* individually or in 1:1 combination produced indels within exon 1 (Figure 2F) and reduced transcript levels (Figure 2G). Combined use of the two vectors was selected as the main strategy because it not only reduced *in vitro* transcript levels, but frequent excision of the intervening 98-bp region between the two sgRNAs additionally disrupted *Aqp*1 function by deleting key protein residues predicted by the crystal structure to affect channel patency (Figures S2A–S2C).[29] No editing was identified for either sgRNA in the top five off-target coding genes predicted by a bioinformatic algorithm (Figure S2D).[30]

Aquaporin 1 Disruption by CRISPR-Cas9 Lowers IOP

Wild-type *C57BL/6J* mice received unilateral intravitreal injection of the combined vector mix, and 3 weeks later the ciliary body was isolated. This confirmed genomic DNA editing within exon 1 of *Aqp1* only in treated eyes (Figure 3A), where the presence of SaCas9 DNA was also detected (Figure 3B). In these mice, IOP was reduced by a mean of 2.9 mmHg relative to each contralateral eye (Figure 3C). This would be approximately a 22% reduction, which would be clinically effective if translated to glaucoma therapy, where trials have shown even a 25% reduction can preserve vision.[31] No statistically significant difference in IOP between eyes was identified at baseline, and greater variance is seen in IOP between different animals, so contralateral eyes were used as the optimal control with datapoints displayed as linked pairs of eyes where indicated (Figure S3). Injection of a GFP-encoding ShH10 control vector did not lead to a reduction in IOP compared with uninjected or MIX-treated eyes (Figure 3D). When intravitreal injection of ShH10 vector was performed in both eyes, subclinical inflammation seen only on OCT scanning as scattered cells within the vitreous cavity could be observed. This was severe in 2 of 23 eyes examined, which were excluded from analysis, and is compatible with loss of local immune regulation secondary to antigen excess as previously observed.[32,33] Flow cytometry of ciliary body from GFP- and MIX-treated eyes, however, did not show differences in infiltrating immune cell numbers to confound the IOP changes seen (Figure S4). Studies imply there is negligible ciliary body epithelium turnover in mammals.[34] BrdU pulse staining was performed in ShH10-injected eyes and did not show secondary epithelial cell proliferation in response to viral infection (Figure S2E).

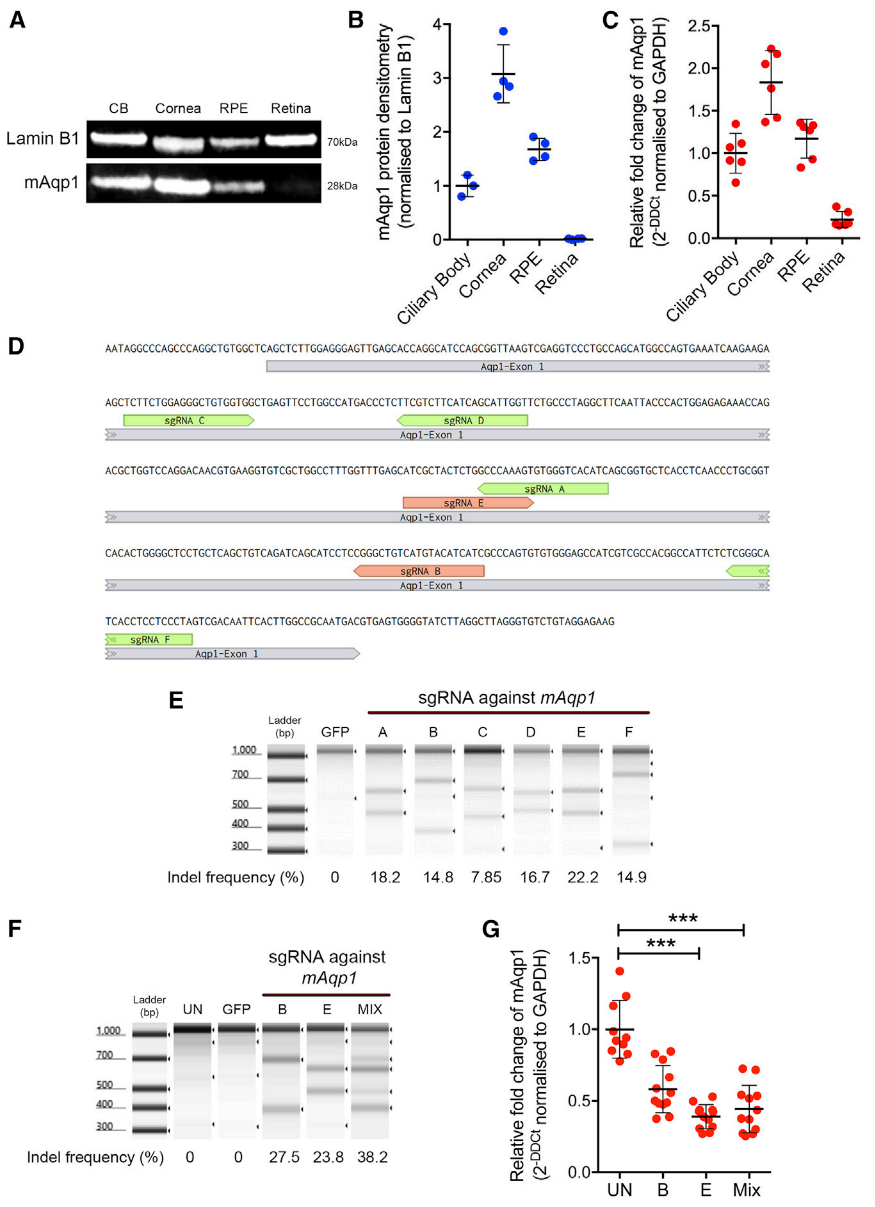

Figure 2. Aquaporin 1 Is Expressed in the Mouse Ciliary Body and Can Be Targeted Using CRISPR-SaCas9

The mouse eye expresses *AQP1* predominantly in the ciliary body, cornea, and RPE. (A–C) Representative western blot and pooled data from dissected tissues (A) for protein (B) and RNA (C) by quantitative PCR; n = 4–6 eyes. (D) Genomic map of exon 1 of mouse *Aqp1* displaying sequence and binding location of tested SaCas9-compatible short guide RNAs (sgRNA) tested. (E) T7 endonuclease 1 assay for different plasmid transfected sgRNAs and the indel creation efficiency of each. sgRNA-labeled B and E were packaged into ShH10 serotype AAV vectors and co-cultured with mouse B6-RPE cells individually and in combination. (F and G) T7 endonuclease 1 assay (F) and quantitative PCR (G) for *mAqp1* were performed. *Aqp1* expression was significantly reduced using sgRNA E alone and in combination with B (MIX) compared with uninfected (UN) controls. Kruskal-Wallis test with Dunn's multiple comparisons. Mean ± SD is shown. ***$p = 0.001$, ****$p < 0.001$. n = 10–12. Lamin B1 was used as loading control.

AQP1 protein levels were reduced in the ciliary body of treated eyes (Figures 3E and 3F). Complete AQP1 loss is not seen because it is not feasible to readily separate the targeted non-pigmented epithelium from other uninfected ciliary body cells. No structural abnormalities in the ciliary body were seen by histology at 3 weeks (Figure 3G), consistent with a lack of gross toxicity reported following retinal delivery of CRISPR-Cas9 variants.[35] Optical coherence tomography (OCT) was used to determine whether any *in vivo* off-target effects from potential *Aqp1* disruption in the cornea or retina occurred (Figure 3H). No edema was clinically apparent, and quantification confirmed that no significant corneal or retinal thickening occurred (Figures 3I and 3J), consistent with previous publications suggesting there is corneal redundancy provided by aquaporin 5.[26]

Aquaporin 1 Disruption Can Prevent Ganglion Cell Loss in Experimental Models of Glaucoma

After demonstrating effective IOP reduction in normal eyes, the approach was trialed in two experimental mouse models of glaucoma. Corticosteroid-induced ocular hypertension produces mildly increased IOP, analogous to the distinct entity seen in humans, but does not lead to robust ganglion cell loss using a depot model.[36] Intervention in this model from an elevated baseline resulted in about 20% reduction in IOP (Figure 4A) and ciliary body *AQP1* protein levels (Figures 4B and 4C).

The microbead glaucoma model produces greater occlusion of aqueous drainage, resulting in markedly elevated IOP and secondary retinal ganglion cell loss. Treatment one week after elevation of IOP led to a sustained reduction (Figure 4D) in mean pressure of 3.9 mmHg at 3 weeks (Figure 4E). *AQP1* protein in the matched eyes was lowered (Figure 4F). Extending the model to 7 weeks demonstrated that treatment prevented the ganglion cell loss (Figures 4G and 4H), illustrating not only IOP reduction but cellular preservation—the definitive aim of any glaucoma therapy.

ShH10 Can Transduce Human Ciliary Body Epithelium

To determine whether the approach could be readily translated into humans, we obtained ocular tissue surplus to transplantation. *AQP*1 protein (Figure 5A) and transcript (Figure 5B) are detected to the same extent in ciliary body and cornea, with minimal

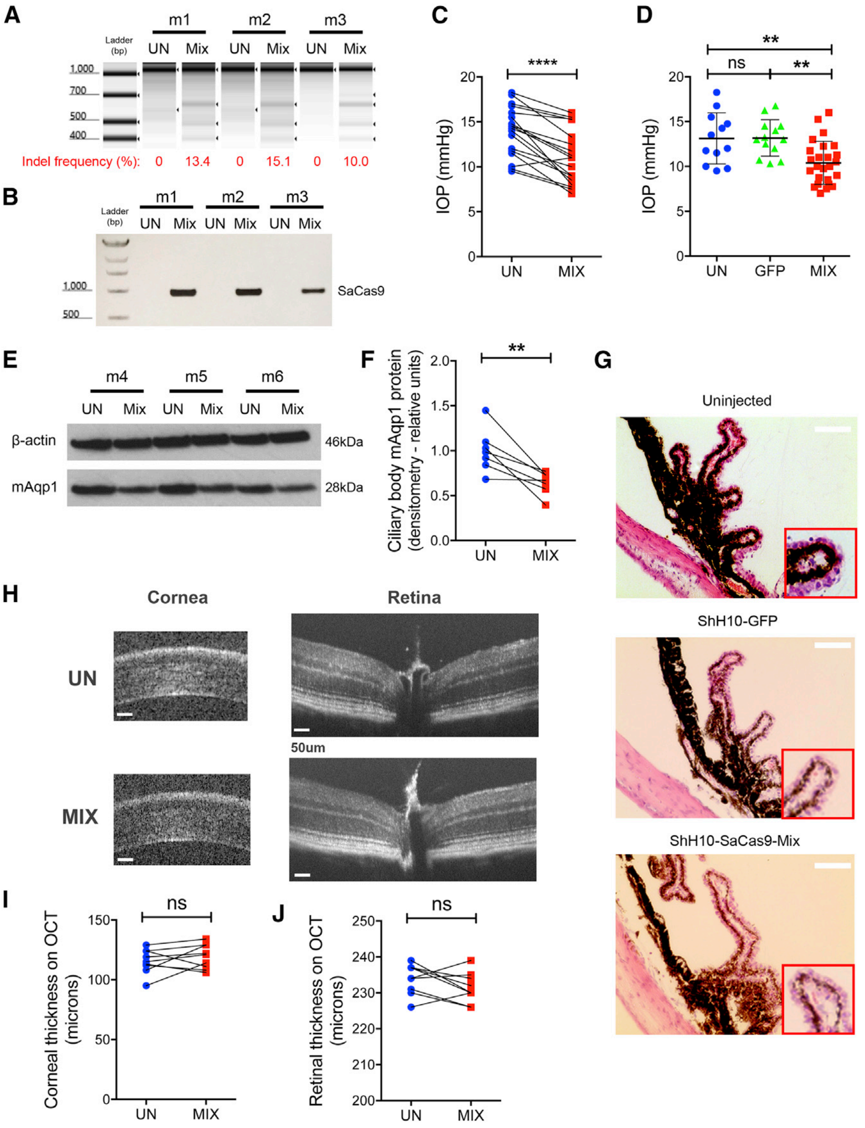

Figure 3. CRISPR-Cas9-Mediated Disruption of Ciliary Body Aquaporin 1 Lowers Intraocular Pressure in the Mouse

Intravitreal injection of 2×10^{10} genome copies of the ShH10 virus encoding an equal proportion of m*Aqp1* B and E sgRNA (MIX) was performed into one eye of each wild-type *C57BL/6J* mouse. After 3 weeks, (A) a representative T7 Endonuclease 1 assay demonstrates genomic DNA mutation in ciliary body dissected from treated eyes, but not in uninjected eyes (UN). (B) SaCas9 DNA is detectable by PCR only in ciliary body tissue from MIX eyes. (C) IOP is reduced by m*Aqp1* disruption by a mean of 2.9 mmHg; paired t test, n = 18 pairs. (D) IOP is not altered by control ShH10 CMV-GFP virus injection, one-way ANOVA with Holm-Sidak's multiple comparison, three independent experiments, n = 50 eyes. (E and F) Representative western blot (E) and densitometry (F) showing reduced *AQP*1 protein in isolated ciliary body tissue; paired t test, n = 7 pairs. (G) Representative H&E-stained paraffin sections of ciliary body show no clear structural abnormalities; n = 6, representative images shown with ×2 original magnification inset. (H) Representative optical coherence tomography (OCT) scans of treated and control eyes. (I and J) No significant "off-target" increase in thickness is seen for either (I) cornea or (J) retina, paired t test, n = 9 pairs. All scale bars: 50 μm. Mean ± SD is shown. $^{**}p = 0.01$, $^{****}p < 0.001$. ns, not significant.

expression in the retina or RPE. The ciliary body was isolated and maintained in culture for up to 7 days according to an established protocol. ShH10 vector encoding GFP was co-incubated and fluorescence was observed *in vitro* from day three onward (Figure 5C) and confirmed as arising from non-pigmented ciliary epithelium by histology at day seven (Figure 5D). sgRNAs aligning to exon 1 of human *AQP1* were tested for their targeting efficiency by plasmid transfection into a 293T cell line (Figure 5E). sgRNA K produced the highest rate of indel formation and so was packaged into the ShH10 vector. Co-incubation with 293T cells (Figure 5F) or human *ex vivo* ciliary body (Figure 5G) produced detectable indel formation in the *AQP1* locus. Low editing levels in the ciliary body likely arise from limitations in maintaining cell viability *ex vivo*, greater dilution of the vector in culture medium, and difficulty in isolating pure non-pigmented ciliary body epithelium from non-transduced cells during preparation for the T7 Endonuclease I assay.

DISCUSSION

By selectively disrupting *Aqp1* in the adult mouse eye, IOP can be reduced from normal physiological baseline or when elevated in experimental models of glaucoma. Proof of concept was achieved using the engineered ShH10 serotype of AAV to deliver the *S. aureus*-derived CRISPR-Cas9 system packaged within a single vector. If translated to humans, this could benefit patients with glaucoma by providing a new treatment that could lower IOP following a single intravitreal injection irrespective of contributory genetic mutations or disease subtype. An approach that uses permanent gene editing is ideal given the chronic nature of glaucoma with persistent IOP elevation over decades.

Contemporary research into new treatments for glaucoma has focused more on techniques to increase aqueous humor outflow.[37,38] Pharmaceutical agents or laser destructive procedures are currently used to reduce inflow at the ciliary body, but their wider use has been limited. Systemic carbonic anhydrase inhibitors such as acetazolamide dramatically reduce IOP but have unacceptable systemic side effects that prevent long-term use. Cyclodiode laser is often employed when other treatments have failed and in cases of severe acute

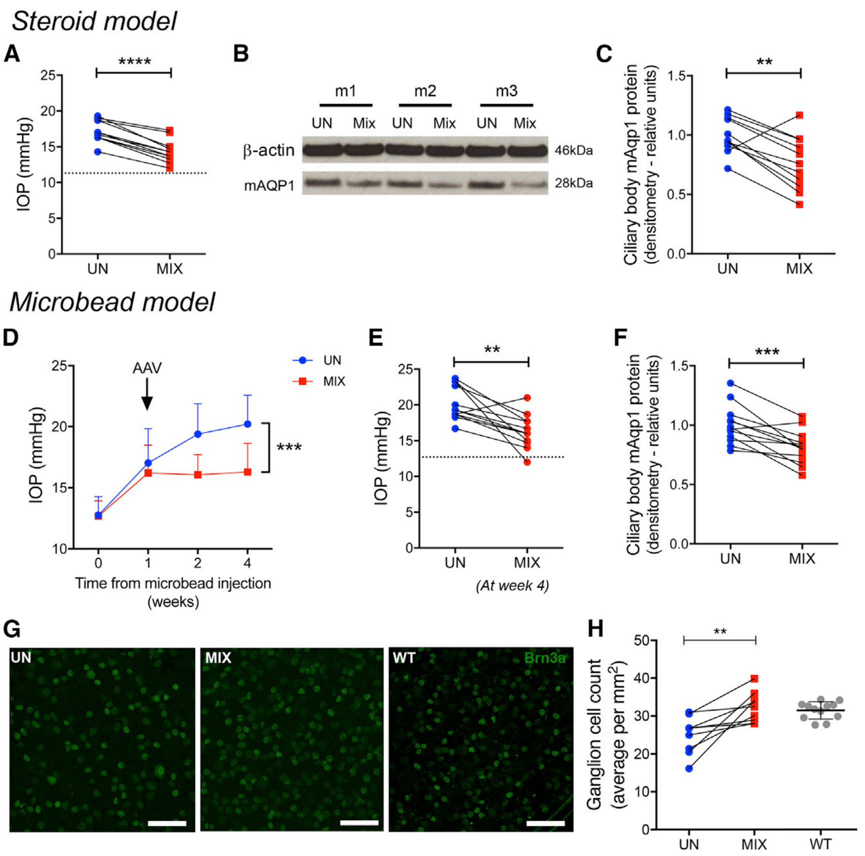

Figure 4. Ciliary Body Aquaporin 1 Disruption Lowers Intraocular Pressure in Two Experimental Glaucoma Models and Prevents Ganglion Cell Loss

Using a corticosteroid-induced ocular hypertension model, paired eyes were subsequently injected with ShH10-CMV-SaCas9-sgRNA B and E (MIX) or untreated (UN). (A) Intraocular pressure (IOP) 3 weeks later is reduced in treated eyes by a mean of 2.88 mmHg; paired t test, n = 11 from two independent experiments. Dotted line is 11.3 mmHg mean IOP before model induction. (B and C) Representative *ex vivo* ciliary body western blot (B) and pooled data (C) demonstrating reduced m*AQP*1 protein levels; paired t test, n = 11. The more acute microbead ocular hypertension model was employed with data shown pooled from three independent experiments of three to five mice per run. (D) Eyes were treated 1 week after microbead injection, which attenuated the increase in IOP. Two-way ANOVA, p = 0.0003, n = 12. (E) After 3 weeks post-virus injection, mean IOP reduction was 3.9 mmHg; paired t test, n = 12. Dotted line represents 12.7 mmHg mean baseline IOP. (F) Matched *ex vivo* ciliary body m*AQP*1 protein was reduced in treated eyes; paired t test, p = 0.0008, n = 12. Extending to 7 weeks, confirmed reduced ganglion cell loss in the treated group. (G and H) Representative examples of retinal flatmount staining for Brn3a (G), with ganglion cell quantification (H), a reference mean Brn3α$^+$ ganglion cell count in wild-type (WT) untreated retina by flatmount, also provided; mean of six fields per eye quantified as mean ± SD per mm^2, two independent experiments; paired t test, n = 9 pairs. Mean ± SD is shown. Scale bars, 50 μm. **p < 0.01, ***p < 0.001, ****p < 0.0001.

glaucoma. It is performed under regional anesthesia, typically in an operating theater, and delivers direct thermocoagulation and necrosis of all constituent cells of the ciliary body. Although several treatment sessions can be required, potent but unpredictable IOP reduction is invariably achieved. Visual loss and phthisis bulbi are not uncommon and are ascribed to excessive IOP reduction but may arguably arise from non-selective destruction and loss of the neurotrophic functions of the ciliary body, which would be left intact by the selective inhibition of aqueous production in this approach.[14]

An advantage over standard gene therapy is that *Aqp1* is a universally expressed protein and its DNA sequence is expected to be broadly conserved among all individuals with glaucoma. No prohibitive personalized medicine approach would be required, unlike gene correction of specific rare mutations. The compartmentalized nature of the eye allows local treatment and limits systemic dissemination of AAV, and the technical simplicity and safety of intravitreal injection permits delivery in a clinic setting, unlike the subretinal route. Combination or adjunctive use with all other treatments including eye drops, laser trabeculoplasty, and trabeculectomy surgery is theoretically feasible and likely to be cumulative in IOP reduction capacity.

Thirteen types of aquaporin channel are recognized in mammals and expressed in the eye, where some redundancy in function exists among them.[25,39] Aquaporin 1 was targeted because it is the most abundant at RNA and protein levels. Aquaporin 4 is also expressed in the ciliary body and might compensate for excessive reduction in IOP following *Aqp1* disruption, in a similar fashion to that observed in the cornea, where aquaporin 5 deficiency is partly compensated for by aquaporin 3.[40]

Several limitations need to be addressed in future work. With the ubiquitous promoter used, all cells transduced by the ShH10 vector will undergo *Aqp1* disruption, including retina and cornea. Although no clear adverse effects were detected, the identification and integration of a ciliary body epithelium-specific promoter or microRNA transcript restriction will be required.[41] Efforts toward this are underway and will be greatly assisted by combining bioinformatic design with transcriptomic profiling. An equal mix of two closely spaced sgRNAs provided consistent IOP reduction and often resulted in excision of the intervening sequence corresponding to a key region of the *Aqp1* channel. This phenomenon has been observed before and although it may not be the final translational approach, it increases the efficiency of disruption of *Aqp1* to confirm proof of concept.[42]

There are disadvantages to the intravitreal route compared with subretinal injection, which was the first to receive FDA approval for AAV ocular gene therapy. Heightened immune responses are

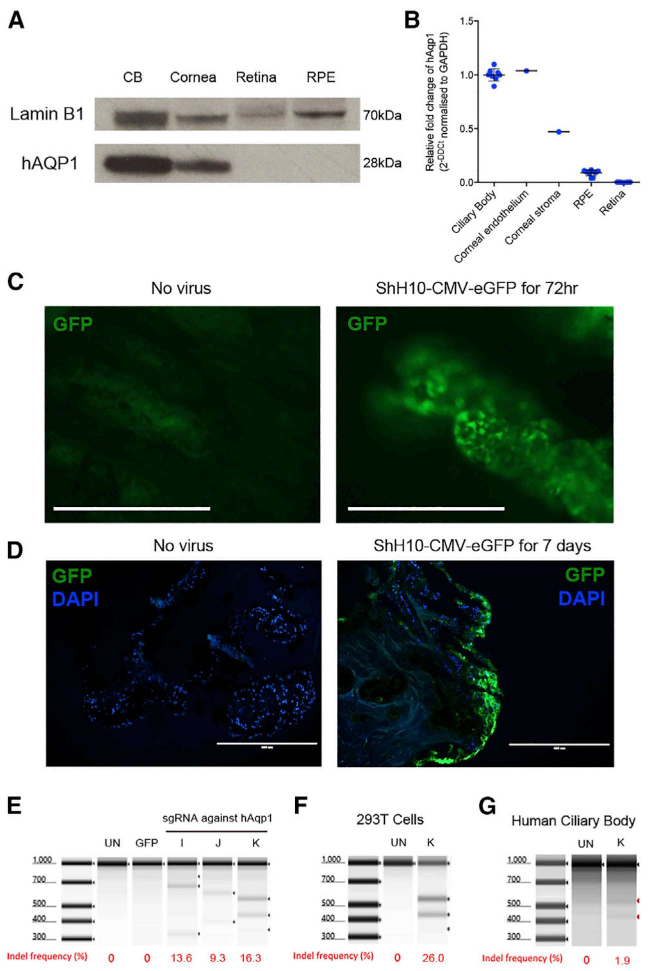

Figure 5. Human Ciliary Body Expresses Aquaporin 1 and Can Be Targeted by ShH10 Vector to Permit CRISPR-Cas9-Mediated Gene Disruption

Human *ex vivo* ciliary body from post-mortem donors was obtained and could be maintained in culture up to 7 days. (A) Representative western blot for aquaporin 1 (h*AQP1*) protein from an eye undergoing immediate dissection. (B) Pooled qPCR expression data of available tissue from several donors, n = 6 (only one globe contained cornea). *AQP1* was enriched in ciliary body and corneal endothelium. Human ciliary body was placed into immediate culture with ShH10 virus expressing GFP under the control of the ubiquitous CMV promoter. (C) By 72 h, GFP expression was detected in the ciliary body epithelium above autofluorescence using live fluorescence microscopy. Identical exposure times were used. (D) At day seven of culture, GFP can be seen in the outer non-pigmented ciliary epithelium using confocal microscopy histological sections. Representative example from four independent cultures. Three human sgRNAs were generated targeting exon 1 of h*AQP1* and (E) tested in 293T cells by plasmid transfection and T7 endonuclease 1 assay. (F) sgRNA K was selected and packaged into an ShH10 vector. Addition to 293T cells for 72 h produced detectable indel formation by T7 endonuclease 1 assay. (G) Co-culture of the same vector with *ex vivo* human ciliary body led to low but detectable indel formation. Mean ± SD is shown. Scale bars, 400 μm.

apparent, with high preexisting neutralizing antibody titers to AAV capsids able to prevent cell infection.[33] In early reports, ocular inflammation has been observed using this route in non-human primate (NHP) studies and early clinical trials.[43–45] We observed possible vitreous infiltrates in a subset of treated eyes, but this was severe only in a small percentage; however, it is likely that for further study the judicious use of immunosuppression and vector refinements will be expedient.

Although ocular inflammation is known to be able to reduce IOP, it is unlikely this explains the therapeutic effect we observed because no reduction is seen with GFP control vector or with a CRISPR-Cas9 vector that does not target *Aqp1* (Figure S3). We also performed flow cytometry of the ciliary body and detected no difference between the lymphoid or myeloid cellular infiltrate of GFP or MIX to confound the IOP reduction observed (Figure S4).

Additional refinements could include the disruption of multiple targets to extend efficacy, such as combining carbonic anhydrases or Connexin 43 disruption with *Aqp1* editing, which are known to also contribute to aqueous humor secretion.[46] The magnitude of IOP reduction achieved by *Aqp1* disruption in the normal eye is highly significant because reduction may be limited to a minimum pressure in the mouse.

Transient or non-viral delivery methods may prove superior to minimize long-term Cas9 expression once gene editing is complete. Other studies have not detected toxicity from persistent Cas9 expression in the retina, however, and because target ocular cells are principally post-mitotic, the risk for increasingly recognized off-target mutations causing subsequent carcinogenesis should remain low.[35,47] AAV is certainly a superior vector and even now FDA approved for ocular gene therapy, compared with other vectors such as adenovirus.[19,23,48]

Further pre-clinical optimization including the use of glaucoma models in large-animal and non-human primate will help to further assess the efficacy and potential side effects of the intervention before proceeding to clinical studies.[49] These will allow accurate measurement of aqueous production rate changes and predicted human IOP response, but experiments would need species-specific sgRNA targeting. Longer-term sequelae and potential immune responses could be studied more precisely and applied in the context of developments emerging in the wider field. Efforts may also be invested in refining *ex vivo* human tissue cultures, with an anterior chamber perfusion method to recapitulate the ocular environment and maintain viability for longer to robustly test permutations of sgRNA.[50] Ultimately, first-in-human trials are required, yet will be aided by the unfortunately high prevalence of patients already suffering complete visual loss from glaucoma but retaining elevated IOP. These poorly sighted eyes would be initial candidates of choice for testing.

Encouraging findings for human translation include the shared tropism of the ShH10 capsid for ciliary body non-pigmented epithelium seen in *ex vivo* ciliary body and detectable genomic editing within the *AQP1* locus by CRISPR-Cas9, even given the limitations of tissue culture maintenance. By harnessing the latest gene-editing and vector advances, this approach has the potential to provide

reduction of IOP after a single intraocular injection, which could ultimately benefit patients with glaucoma if successfully translated.

MATERIALS AND METHODS

Animal Husbandry

Adult (6- to 8-week-old) *C57BL/6J* female mice were purchased from Charles River Laboratories, Oxford, UK, and housed at the University of Bristol Animal Services Unit under specific pathogen-free conditions with food and water *ad libitum*. All procedures were conducted in concordance with the United Kingdom Home Office licenses (PPL 30/3045 and 30/3281) and were approved by the University of Bristol Ethical Review Group. The study also complied with the Association for Research in Vision and Ophthalmology (ARVO) statement for the use of animals in ophthalmic and vision research.

IOP Measurement

Mice were anesthetized using 2.5% isoflurane in pure oxygen, and IOP was immediately measured using the TonoLab rebound tonometer (Icare, Vantaa, Finland) according to the manufacturer's instructions. IOP measurement was standardized to be performed between 16:00 and 18:00 GMT. Eyes were alternately tested after 3 min following induction of anesthesia. The mean of three successful measurements for each eye was used for analysis, with each TonoLab measurement comprising an average of six rebound tests. Paired eyes were tested alternately to control for anesthetic effects on IOP.

Intravitreal Injection and *In Vivo* Imaging

Mice were anesthetized using an intraperitoneal injection of 90 μL/10 g body weight of a solution of Ketavet (ketamine hydrochloride 100 mg/mL; Zoetis Ireland, Dublin, Ireland) and Rompun (xylazine hydrochloride 20 mg/mL; Bayer PLC, Newbury, UK) mixed with sterile water in the ratio of 0.6:1:8.4, respectively.

All intravitreal injections used were 2 μL in volume at the titers indicated in the text and were delivered using an operating microscope and a 33G needle on a microsyringe under direct visualization (Hamilton Company, Reno, NV, USA). 1% Chloramphenicol ointment (Martindale Pharma, Wooburn Green, UK) was applied topically immediately following injection. Pupils were dilated with a single drop of 1% w/v Tropicamide (Chauvin Pharmaceuticals, Romford, UK) prior to fluorescent retinal imaging or OCT of corneal and retinal thickness using the Micron IV platform (Phoenix Research Laboratories, Pleasanton, CA, USA).

Experimental Mouse Models of Ocular Hypertension

The corticosteroid-induced model of ocular hypertension was performed as previously described using Dexamethasone-21-acetate in a vehicle suspension formulation.[36] Two hundred micrograms was injected into the periocular space of both eyes with a 33G needle and Hamilton microsyringe every 7 days for the duration of each experiment. The inclusion criteria were successful sub-conjunctival injection and elevated IOP 1 week after induction compared with baseline. Allocation of eyes to intervention was made at random.

The microbead ocular hypertension model followed a published protocol.[51] In brief, after anesthesia and pupil dilation, 3×10^6 sterile paramagnetic microbeads of 4.5 μm in diameter (Dynabeads, Thermo Fisher Scientific, UK) were injected into the anterior chamber using a 70-μm internal diameter borosilicate glass micropipette and microsyringe pump (World Precision Instruments, Hitchin, UK). A 0.45-T bar magnet was used to direct beads into the angle and 1% chloramphenicol ointment applied as above.

IOP was checked before injection or model induction. AAV treatment was randomly distributed to eyes with one as intervention and the other as a contralateral control. AAV injection was given 1 week after induction of the ocular hypertension model.

Cell Lines and Tissue Culture

Mouse B6-RPE07 (gift of Dr. Heping Xu, Queen's University Belfast, Belfast, UK) and human 293T cell lines (ATCC CRL-3216) were maintained in culture using DMEM supplemented with 10% v/v heat-inactivated fetal calf serum, 2 mM L-glutamine, 1 mM sodium pyruvate, 100 U/mL penicillin, and 100 μg/mL streptomycin (all from Thermo Fisher Scientific, UK).[52] Cells were incubated at 37°C in 5% CO_2 using standard tissue culture conditions, passaged twice per week a maximum of 20 times. Transient plasmid transfection was performed using Lipofectamine 3000 and Opti-MEM medium (Thermo Fisher Scientific, UK), according to the manufacturer's instructions, on cells at 70% confluency and incubated for 72 h.

Design of Mouse and Human sgRNA Sequences

The mouse *Aqp1* genomic sequence was obtained from Ensembl (ENSMUSG00000004655). Compatible 21-bp SaCas9 sgRNAs were identified and ranked using Benchling (https://benchling.com) according to Doench et al.[53] Selected sgRNAs were synthesized as oligonucleotides (Sigma Aldrich, UK) and cloned into the pX601-AAV-CMV:NLS-SaCas9-NLS-3xHA-bGHpA;U6::BsaI-sgRNA plasmid from Addgene by Golden Gate assembly according to the supplied protocol.[22] The top five off-target coding genes for each sgRNA were also determined for testing as per Bae et al.[30]

AAV Production

AAV vectors were either purchased from Vector Biolabs (PA, USA) or manufactured at the UCL (University College London) Institute of Ophthalmology as previously published.[54] In brief, recombinant ShH10 serotype particles were produced through triple-plasmid transfection using PEI transfection reagent into 293T-HEK cells. ShH10 particles were bound to a 1-mL HiTrap AVB Sepharose column (GE Healthcare, USA) and eluted with 50 mM glycine (pH 2.7) into 1 M Tris (pH 8.8). Vectors were desalted and concentrated in PBS-MK to a concentration of 1×10^{13} genome copies per milliliter (gc/mL) using a Vivaspin 4 (10 kDa) concentrator. Viral genome titers were determined by quantitative real-time PCR using probes binding to either the SV40 or ITR sequences. An amplicon-based standard series of known concentration was used for sample interpolation. Preparations were certified as endotoxin <5 EU/mL by

Pyrotell-T kinetic turbidimetric endotoxin test (Associates of Cape Cod, MA, USA).

T7 Endonuclease 1 Genomic Cleavage Assay

Genomic DNA was extracted from cells and tissue using DNeasy Blood & Tissue Kit (QIAGEN, Germany). The region of expected indel formation was amplified by PCR asymmetrically spanning the region using the Q5 High-Fidelity DNA polymerase master mix (New England BioLabs, MA, USA) with primers shown in Supplemental Information. The PCR product was denatured and reannealed before cleavage with T7 endonuclease 1 (New England BioLabs, MA, USA) according to the manufacturer's instructions. Fragments were analyzed by a DNA 1000 assay on an Agilent 2100 Bioanalyzer (Agilent Technologies, CA, USA) with indel formation frequency determined as per Ran et al.[55]

RNA Isolation and qPCR

Total mRNA was isolated using the RNeasy Mini Kit (QIAGEN, Germany) before a RNA to Ct one-step TaqMan assay was performed on a QuantStudio 3 (Thermo Fisher Scientific, UK) as per the manufacturer's protocol. The TaqMan *Aqp1* probes used were Mm01326466_m1 and Hs00166067_m1, and GAPDH probes were Mm99999915_g1 and Hs02758991_g1 (Thermo Fisher Scientific, UK). See Supplemental Information for probes and primers sequences. Each sample was run in triplicate and normalized against GAPDH using the $2^{-\Delta\Delta Ct}$ method.[56]

Western Blot Analysis

Protein was extracted from cells or tissues using the Cellytic MT lysis reagent (Sigma-Aldrich, UK), and protein concentration was determined by the BCA protein assay kit (Thermo Fisher Scientific, UK). Between 10 and 40 μg total protein was prepared per sample and denatured using Bolt LDS sample buffer (Thermo Fisher Scientific, UK). Electrophoresis was performed on precast 4%–12% Bis-Tris Plus gels before transfer to a polyvinylidene fluoride (PVDF) membrane using the iBlot system (Thermo Fisher Scientific, UK). After blocking for 1 h with 5% skimmed milk in 0.1% TBS-Tween, the membrane was stained overnight at 4°C with 1:1,000 dilution of primary antibody, either anti-mouse/human *AQP*1 (ab168387; Abcam, Cambridge, UK), anti-β-actin (4970; Cell Signaling Technology, MA, USA), or anti-Lamin B1 (ab133741; Abcam, Cambridge, UK). After washing, the membrane was incubated with either HRP (7074; Cell Signaling Technology, MA, USA) or DyLight 800 (SA5-10036; Thermo Fisher Scientific, UK)-conjugated secondary antibodies and developed with ECL Prime reagent (RPN2232; GE Healthcare, USA) and film or the LI-COR Odyssey Fc imaging system (LI-COR Biosciences, NE, USA).

Histology

Mouse eyes were dissected and fixed in 2% paraformaldehyde before paraffin embedding, sectioning at 6-μm thickness, and hematoxylin and eosin staining at the Histology Core Facility, University of Bristol. Images were captured using an EVOS Color CCD microscope (Thermo Fisher Scientific, UK).

For GFP immunofluorescent imaging, mouse eyes or human ciliary body was fixed with 4% paraformaldehyde, frozen in optical cutting temperature compound (VWR, PA, USA), and sectioned at 14-μm intervals. Slides were incubated with a 1:1,000 dilution of DAPI (Sigma Aldrich, UK) and mounted in fluorescence mounting media (Agilent Technologies, CA, USA) before imaging on an EVOS FL microscope (Thermo Fisher Scientific, UK) or Leica SP5 Confocal microscope (Leica Microsystems, Germany).

For ganglion cell counts, mouse retinal flatmounts were prepared by immediate dissection as previously described and fixed in 4% PFA for 2 h.[57] Tissues were blocked in 5% normal goat serum (Vector Laboratories, CA, USA), 1% Triton X-100, and 2% BSA (Sigma Aldrich, UK) in PBS for 4 h before 2-day incubation at 4°C with 1:50 dilution of Alexa Fluor 488-conjugated anti-mouse Brn-3α (sc-8429; Santa Cruz Biotechnology, TX, USA). Five z stacks encompassing the ganglion cell layer distributed evenly around the optic disc were imaged at ×63 magnification using a SP5 confocal laser scanning microscope (Leica Microsystems, Germany). Using maximum projection images, mean Brn-3α retinal ganglion cell counts were quantified with Volocity (Version 6.2.1; Perkin Elmer, UK). The registration module was used to obtain an average count per field of positive cells with diameter between 10 and 40 μm.

Human Ciliary Body Culture

Human donor eye material surplus to corneal transplantation (without recorded ocular disease) was obtained from National Health Service (NHS) Blood and Transplant Services after research ethics committee approval (16/SW/0124), with experiments conducted according to the Declaration of Helsinki and in compliance with UK law. Dissected human ciliary body processes were immediately placed into epithelial cell culture medium (ScienCell Research Laboratories, Carlsbad, CA, USA) incubated at 37°C and 5% CO_2 comparable with a previously published protocol for a maximum of 7 days.[58] Where indicated, culture media were supplemented with ShH10 vector to a concentration of 1.5×10^{11} gc/mL.

Statistics

Results are presented as mean ± standard deviation (SD) in all cases. Comparisons of two individual groups were performed using either paired or unpaired Student's t test and Mann-Whitney test. For multiple comparisons, nonparametric analysis was performed using the Kruskal-Wallis with Dunn's multiple comparisons test. Tests were performed on GraphPad Prism 6 (v.6.01; GraphPad Software, CA, USA). Two-tailed tests were used throughout, and results were considered statistically significant if $p < 0.05$.

SUPPLEMENTAL INFORMATION

Supplemental Information can be found online at https://doi.org/10.1016/j.ymthe.2019.12.012.

AUTHOR CONTRIBUTIONS

J.W., O.H.B., D.A.C., A.Y., J.R.P., R.M., R.S.E., and C.J.C. conducted the experiments. J.W., C.J.C., D.A.C., and A.D.D. designed the

experiments, analyzed the data, and drafted the paper. C.J.C., R.R.A., P.T.K., and A.D.D. provided study supervision and technical, administrative, and material support. C.J.C. conceived the project. All authors edited and approved the final manuscript.

CONFLICTS OF INTEREST

The authors declare no competing interests.

ACKNOWLEDGMENTS

This work was supported by grant funding from the National Eye Research Centre (BRI 019), the T.F.C. Frost Charitable Trust, Above & Beyond, Bristol, Fight for Sight (1730/1731), the Elizabeth Blackwell Institute for Health Research, the University of Bristol, and the Medical Research Council. Support was provided to P.T.K., R.R.A., and A.D.D. by the National Institute for Health Research (NIHR) Biomedical Research Centre based at Moorfields Eye Hospital NHS Foundation Trust and UCL Institute of Ophthalmology. R.R.A. was also supported by Retina UK. C.J.C. was supported as an NIHR Academic Clinical Lecturer. The views expressed are those of the authors and not necessarily those of the NHS, the NIHR, or the Department of Health. A patent relating to this work has been filed by the University of Bristol. We wish to acknowledge the Wolfson Bioimaging Facility and the Genomics Facility at the University of Bristol for their assistance. The B6-RPE07 cell line was a gift of Dr. Heping Xu, Queen's University Belfast, Belfast, UK. Shh10 plasmid was a gift from John Flannery & David Schaffer (Addgene plasmid #64867). pX601-AAV-CMV::NLS-SaCas9-NLS-3xHA-bGHpA;U6::BsaI-sgRNA plasmid was a gift from Feng Zhang (Addgene plasmid #61591).[22]

REFERENCES

1. Tham, Y.-C., Li, X., Wong, T.Y., Quigley, H.A., Aung, T., and Cheng, C.-Y. (2014). Global prevalence of glaucoma and projections of glaucoma burden through 2040: a systematic review and meta-analysis. Ophthalmology *121*, 2081–2090.

2. Garway-Heath, D.F., Crabb, D.P., Bunce, C., Lascaratos, G., Amalfitano, F., Anand, N., Azuara-Blanco, A., Bourne, R.R., Broadway, D.C., Cunliffe, I.A., et al. (2015). Latanoprost for open-angle glaucoma (UKGTS): a randomised, multicentre, placebo-controlled trial. Lancet *385*, 1295–1304.

3. Collaborative Normal-Tension Glaucoma Study Group (1998). The effectiveness of intraocular pressure reduction in the treatment of normal-tension glaucoma. Am. J. Ophthalmol. *126*, 498–505.

4. Weinreb, R.N., and Khaw, P.T. (2004). Primary open-angle glaucoma. Lancet *363*, 1711–1720.

5. Mokhles, P., Schouten, J.S., Beckers, H.J., Azuara-Blanco, A., Tuulonen, A., and Webers, C.A. (2016). A Systematic Review of End-of-Life Visual Impairment in Open-Angle Glaucoma: An Epidemiological Autopsy. J. Glaucoma *25*, 623–628.

6. Nordstrom, B.L., Friedman, D.S., Mozaffari, E., Quigley, H.A., and Walker, A.M. (2005). Persistence and adherence with topical glaucoma therapy. Am. J. Ophthalmol. *140*, 598–606.

7. Schwartz, G.F., and Quigley, H.A. (2008). Adherence and persistence with glaucoma therapy. Surv. Ophthalmol. *53* (*Suppl 1*), S57–S68.

8. Kirwan, J.F., Lockwood, A.J., Shah, P., Macleod, A., Broadway, D.C., King, A.J., McNaught, A.I., and Agrawal, P.; Trabeculectomy Outcomes Group Audit Study Group (2013). Trabeculectomy in the 21st century: a multicenter analysis. Ophthalmology *120*, 2532–2539.

9. Landers, J., Martin, K., Sarkies, N., Bourne, R., and Watson, P. (2012). A twenty-year follow-up study of trabeculectomy: risk factors and outcomes. Ophthalmology *119*, 694–702.

10. Gedde, S.J., Herndon, L.W., Brandt, J.D., Budenz, D.L., Feuer, W.J., and Schiffman, J.C.; Tube Versus Trabeculectomy Study Group (2012). Postoperative complications in the Tube Versus Trabeculectomy (TVT) study during five years of follow-up. Am. J. Ophthalmol. *153*, 804–814.e1.

11. Braunger, B.M., Fuchshofer, R., and Tamm, E.R. (2015). The aqueous humor outflow pathways in glaucoma: A unifying concept of disease mechanisms and causative treatment. Eur. J. Pharm. Biopharm. *95*, 173–181.

12. Rotchford, A.P., Jayasawal, R., Madhusudhan, S., Ho, S., King, A.J., and Vernon, S.A. (2010). Transscleral diode laser cycloablation in patients with good vision. Br. J. Ophthalmol. *94*, 1180–1183.

13. Murphy, C.C., Burnett, C.A., Spry, P.G., Broadway, D.C., and Diamond, J.P. (2003). A two centre study of the dose-response relation for transscleral diode laser cyclophotocoagulation in refractory glaucoma. Br. J. Ophthalmol. *87*, 1252–1257.

14. Coca-Prados, M., and Escribano, J. (2007). New perspectives in aqueous humor secretion and in glaucoma: the ciliary body as a multifunctional neuroendocrine gland. Prog. Retin. Eye Res. *26*, 239–262.

15. Borrás, T., Buie, L.K., and Spiga, M.G. (2016). Inducible scAAV2.GRE.MMP1 lowers IOP long-term in a large animal model for steroid-induced glaucoma gene therapy. Gene Ther. *23*, 438–449.

16. O'Callaghan, J., Crosbie, D.E., Cassidy, P.S., Sherwood, J.M., Flügel-Koch, C., Lütjen-Drecoll, E., Humphries, M.M., Reina-Torres, E., Wallace, D., Kiang, A.S., et al. (2017). Therapeutic potential of AAV-mediated MMP-3 secretion from corneal endothelium in treating glaucoma. Hum. Mol. Genet. *26*, 1230–1246.

17. Osborne, A., Khatib, T.Z., Songra, L., Barber, A.C., Hall, K., Kong, G.Y.X., Widdowson, P.S., and Martin, K.R. (2018). Neuroprotection of retinal ganglion cells by a novel gene therapy construct that achieves sustained enhancement of brain-derived neurotrophic factor/tropomyosin-related kinase receptor-B signaling. Cell Death Dis. *9*, 1007.

18. Martin, K.R., Quigley, H.A., Zack, D.J., Levkovitch-Verbin, H., Kielczewski, J., Valenta, D., Baumrind, L., Pease, M.E., Klein, R.L., and Hauswirth, W.W. (2003). Gene therapy with brain-derived neurotrophic factor as a protection: retinal ganglion cells in a rat glaucoma model. Invest. Ophthalmol. Vis. Sci. *44*, 4357–4365.

19. Jain, A., Zode, G., Kasetti, R.B., Ran, F.A., Yan, W., Sharma, T.P., Bugge, K., Searby, C.C., Fingert, J.H., Zhang, F., et al. (2017). CRISPR-Cas9-based treatment of myocilin-associated glaucoma. Proc. Natl. Acad. Sci. USA *114*, 11199–11204.

20. Khawaja, A.P., Cooke Bailey, J.N., Wareham, N.J., Scott, R.A., Simcoe, M., Igo, R.P., Jr., Song, Y.E., Wojciechowski, R., Cheng, C.Y., Khaw, P.T., et al.; UK Biobank Eye and Vision Consortium; NEIGHBORHOOD Consortium (2018). Genome-wide analyses identify 68 new loci associated with intraocular pressure and improve risk prediction for primary open-angle glaucoma. Nat. Genet. *50*, 778–782.

21. Abu-Amero, K., Kondkar, A.A., and Chalam, K.V. (2015). An Updated Review on the Genetics of Primary Open Angle Glaucoma. Int. J. Mol. Sci. *16*, 28886–28911.

22. Ran, F.A., Cong, L., Yan, W.X., Scott, D.A., Gootenberg, J.S., Kriz, A.J., Zetsche, B., Shalem, O., Wu, X., Makarova, K.S., et al. (2015). In vivo genome editing using Staphylococcus aureus Cas9. Nature *520*, 186–191.

23. Russell, S., Bennett, J., Wellman, J.A., Chung, D.C., Yu, Z.-F.F., Tillman, A., Wittes, J., Pappas, J., Elci, O., McCague, S., et al. (2017). Efficacy and safety of voretigene neparvovec (AAV2-hRPE65v2) in patients with RPE65-mediated inherited retinal dystrophy: a randomised, controlled, open-label, phase 3 trial. Lancet *390*, 849–860.

24. Verkman, A.S., and Mitra, A.K. (2000). Structure and function of aquaporin water channels. Am. J. Physiol. Renal Physiol. *278*, F13–F28.

25. Schey, K.L., Wang, Z., L Wenke, J., and Qi, Y. (2014). Aquaporins in the eye: expression, function, and roles in ocular disease. Biochim. Biophys. Acta *1840*, 1513–1523.

26. Zhang, D., Vetrivel, L., and Verkman, A.S. (2002). Aquaporin deletion in mice reduces intraocular pressure and aqueous fluid production. J. Gen. Physiol. *119*, 561–569.

27. Jinek, M., Chylinski, K., Fonfara, I., Hauer, M., Doudna, J.A., and Charpentier, E. (2012). A programmable dual-RNA-guided DNA endonuclease in adaptive bacterial immunity. Science *337*, 816–821.

28. Zhang, J.P., Li, X.L., Li, G.H., Chen, W., Arakaki, C., Botimer, G.D., Baylink, D., Zhang, L., Wen, W., Fu, Y.W., et al. (2017). Efficient precise knockin with a double

cut HDR donor after CRISPR/Cas9-mediated double-stranded DNA cleavage. Genome Biol. *18*, 35.

29. Ren, G., Reddy, V.S., Cheng, A., Melnyk, P., and Mitra, A.K. (2001). Visualization of a water-selective pore by electron crystallography in vitreous ice. Proc. Natl. Acad. Sci. USA *98*, 1398–1403.

30. Bae, S., Park, J., and Kim, J.-S. (2014). Cas-OFFinder: a fast and versatile algorithm that searches for potential off-target sites of Cas9 RNA-guided endonucleases. Bioinformatics *30*, 1473–1475.

31. Heijl, A., Leske, M.C., Bengtsson, B., Hyman, L., Bengtsson, B., Hussein, M., and Early Manifest Glaucoma Trial Group. (2002). Reduction of intraocular pressure and glaucoma progression: results from the Early Manifest Glaucoma Trial. Arch Ophthalmol. *120*, 1268–1279.

32. Li, Q., Miller, R., Han, P.-Y.Y., Pang, J., Dinculescu, A., Chiodo, V., and Hauswirth, W.W. (2008). Intraocular route of AAV2 vector administration defines humoral immune response and therapeutic potential. Mol. Vis. *14*, 1760–1769.

33. Kotterman, M.A., Yin, L., Strazzeri, J.M., Flannery, J.G., Merigan, W.H., and Schaffer, D.V. (2015). Antibody neutralization poses a barrier to intravitreal adeno-associated viral vector gene delivery to non-human primates. Gene Ther. *22*, 116–126.

34. McDonald, T.F., and Green, K. (1988). Cell turnover in ciliary epithelium compared to other slow renewing epithelia in the adult mouse. Curr. Eye Res. *7*, 247–252.

35. Jo, D.H., Koo, T., Cho, C.S., Kim, J.H., Kim, J.S., and Kim, J.H. (2019). Long-Term Effects of In Vivo Genome Editing in the Mouse Retina Using Campylobacter jejuni Cas9 Expressed via Adeno-Associated Virus. Mol. Ther. *27*, 130–136.

36. Patel, G.C., Phan, T.N., Maddineni, P., Kasetti, R.B., Millar, J.C., Clark, A.F., and Zode, G.S. (2017). Dexamethasone-Induced Ocular Hypertension in Mice: Effects of Myocilin and Route of Administration. Am. J. Pathol. *187*, 713–723.

37. Lavia, C., Dallorto, L., Maule, M., Ceccarelli, M., and Fea, A.M. (2017). Minimally-invasive glaucoma surgeries (MIGS) for open angle glaucoma: A systematic review and meta-analysis. PLoS ONE *12*, e0183142.

38. Conlon, R., Saheb, H., and Ahmed, I.I. (2017). Glaucoma treatment trends: a review. Can. J. Ophthalmol. *52*, 114–124.

39. Verkman, A.S., Ruiz-Ederra, J., and Levin, M.H. (2008). Functions of aquaporins in the eye. Prog. Retin. Eye Res. *27*, 420–433.

40. Levin, M.H., and Verkman, A.S. (2004). Aquaporin-dependent water permeation at the mouse ocular surface: in vivo microfluorimetric measurements in cornea and conjunctiva. Invest. Ophthalmol. Vis. Sci. *45*, 4423–4432.

41. Qiao, C., Yuan, Z., Li, J., He, B., Zheng, H., Mayer, C., Li, J., and Xiao, X. (2011). Liver-specific microRNA-122 target sequences incorporated in AAV vectors efficiently inhibits transgene expression in the liver. Gene Ther. *18*, 403–410.

42. Tabebordbar, M., Zhu, K., Cheng, J.K.W., Chew, W.L., Widrick, J.J., Yan, W.X., Maesner, C., Wu, E.Y., Xiao, R., Ran, F.A., et al. (2016). In vivo gene editing in dystrophic mouse muscle and muscle stem cells. Science *351*, 407–411.

43. Heier, J.S., Kherani, S., Desai, S., Dugel, P., Kaushal, S., Cheng, S.H., Delacono, C., Purvis, A., Richards, S., Le-Halpere, A., et al. (2017). Intravitreous injection of AAV2-sFLT01 in patients with advanced neovascular age-related macular degeneration: a phase 1, open-label trial. Lancet *390*, 50–61.

44. Cukras, C., Wiley, H.E., Jeffrey, B.G., Sen, H.N., Turriff, A., Zeng, Y., Vijayasarathy, C., Marangoni, D., Ziccardi, L., Kjellstrom, S., et al. (2018). Retinal AAV8-RS1 Gene Therapy for X-Linked Retinoschisis: Initial Findings from a Phase I/IIa Trial by Intravitreal Delivery. Mol. Ther. *26*, 2282–2294.

45. Day, T.P., Byrne, L.C., Flannery, J.G., and Schaffer, D.V. (2018). Screening for Neutralizing Antibodies Against Natural and Engineered AAV Capsids in Nonhuman Primate Retinas. Methods Mol. Biol. *1715*, 239–249.

46. Calera, M.R., Wang, Z., Sanchez-Olea, R., Paul, D.L., Civan, M.M., and Goodenough, D.A. (2009). Depression of intraocular pressure following inactivation of connexin43 in the nonpigmented epithelium of the ciliary body. Invest. Ophthalmol. Vis. Sci. *50*, 2185–2193.

47. Kosicki, M., Tomberg, K., and Bradley, A. (2018). Repair of double-strand breaks induced by CRISPR-Cas9 leads to large deletions and complex rearrangements. Nat. Biotechnol. *36*, 765–771.

48. Cotter, M.J., and Muruve, D.A. (2005). The induction of inflammation by adenovirus vectors used for gene therapy. Front. Biosci. *10*, 1098–105.

49. Rasmussen, C.A., and Kaufman, P.L. (2005). Primate glaucoma models. J. Glaucoma *14*, 311–314.

50. Camras, L.J., Yuan, F., Fan, S., Samuelson, T.W., Ahmed, I.K., Schieber, A.T., and Toris, C.B. (2012). A novel Schlemm's Canal scaffold increases outflow facility in a human anterior segment perfusion model. Invest. Ophthalmol. Vis. Sci. *53*, 6115–6121.

51. Ito, Y.A., Belforte, N., Cueva Vargas, J.L., and Di Polo, A. (2016). A Magnetic Microbead Occlusion Model to Induce Ocular Hypertension-Dependent Glaucoma in Mice. J. Vis. Exp. *109*, e53731.

52. Chen, M., Muckersie, E., Robertson, M., Fraczek, M., Forrester, J.V., and Xu, H. (2008). Characterization of a spontaneous mouse retinal pigment epithelial cell line B6-RPE07. Invest. Ophthalmol. Vis. Sci. *49*, 3699–3706.

53. Doench, J.G., Fusi, N., Sullender, M., Hegde, M., Vaimberg, E.W., Donovan, K.F., Smith, I., Tothova, Z., Wilen, C., Orchard, R., et al. (2016). Optimized sgRNA design to maximize activity and minimize off-target effects of CRISPR-Cas9. Nat. Biotechnol. *34*, 184–191.

54. Gonzalez-Cordero, A., Goh, D., Kruczek, K., Naeem, A., Fernando, M., Kleine Holthaus, S.M., Takaaki, M., Blackford, S.J.I., Kloc, M., Agundez, L., et al. (2018). Assessment of AAV Vector Tropisms for Mouse and Human Pluripotent Stem Cell-Derived RPE and Photoreceptor Cells. Hum. Gene Ther. *29*, 1124–1139.

55. Ran, F.A., Hsu, P.D., Wright, J., Agarwala, V., Scott, D.A., and Zhang, F. (2013). Genome engineering using the CRISPR-Cas9 system. Nat. Protoc. *8*, 2281–2308.

56. Livak, K.J., and Schmittgen, T.D. (2001). Analysis of relative gene expression data using real-time quantitative PCR and the 2(-Delta Delta C(T)) Method. Methods *25*, 402–408.

57. Chu, C.J., Herrmann, P., Carvalho, L.S., Liyanage, S.E., Bainbridge, J.W., Ali, R.R., Dick, A.D., and Luhmann, U.F. (2013). Assessment and in vivo scoring of murine experimental autoimmune uveoretinitis using optical coherence tomography. PLoS ONE *8*, e63002.

58. Eichhorn, M., Inada, K., and Lütjen-Drecoll, E. (1991). Human ciliary body in organ culture. Curr. Eye Res. *10*, 277–286.

YMTHE, Volume *28*

Supplemental Information

Gene Therapy for Glaucoma by Ciliary Body Aquaporin 1 Disruption Using CRISPR-Cas9

Jiahui Wu, Oliver H. Bell, David A. Copland, Alison Young, John R. Pooley, Ryea Maswood, Rachel S. Evans, Peng Tee Khaw, Robin R. Ali, Andrew D. Dick, and Colin J. Chu

Supplemental Figures:

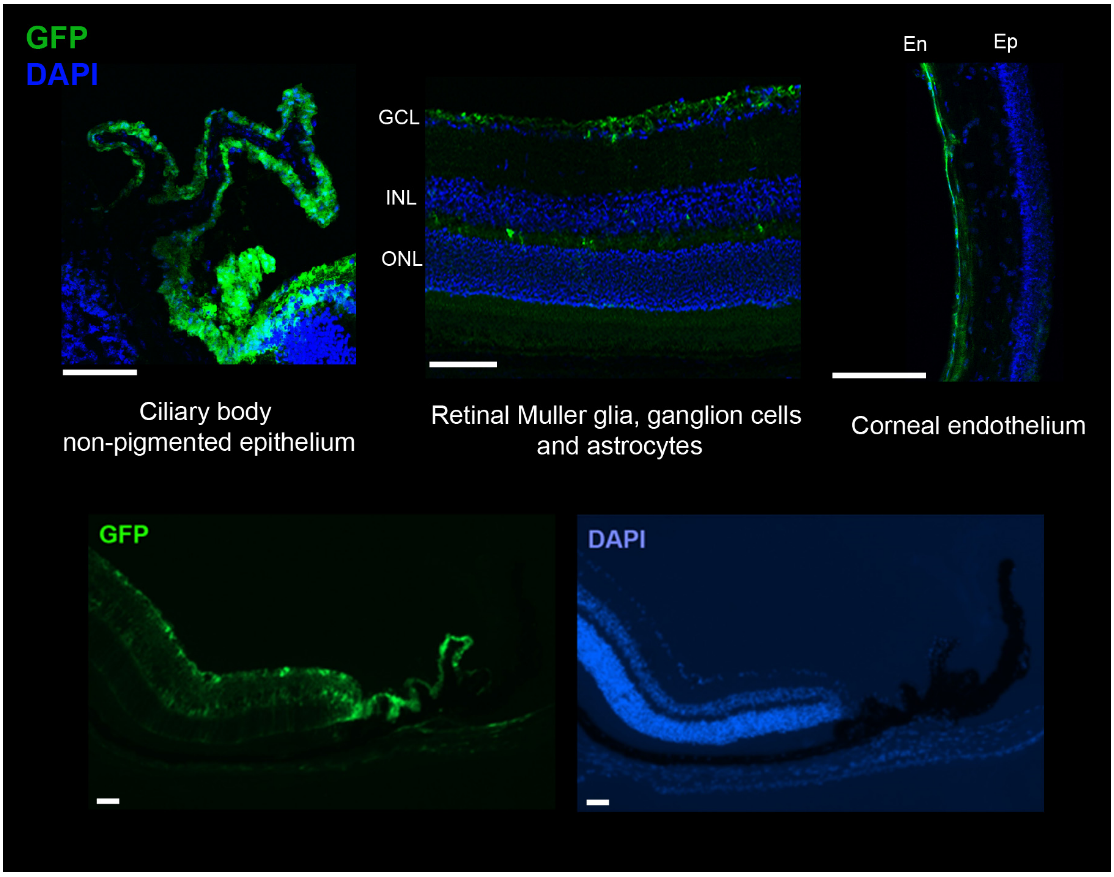

Figure S1. ShH10 produces transduction of ciliary body non-pigmented epithelium, retinal astrocytes, Müller glia, ganglion cells and corneal endothelium. Representative confocal images of *C57BL/6J* mouse eyes four weeks following intravitreal injection of 2×10^{10} genome copies of ShH10-CMV-eGFP. GCL: ganglion cell layer, INL: inner nuclear layer, ONL: outer nuclear layer, En: corneal endothelium, Ep: corneal epithelium. Scale bars: 100 µm.

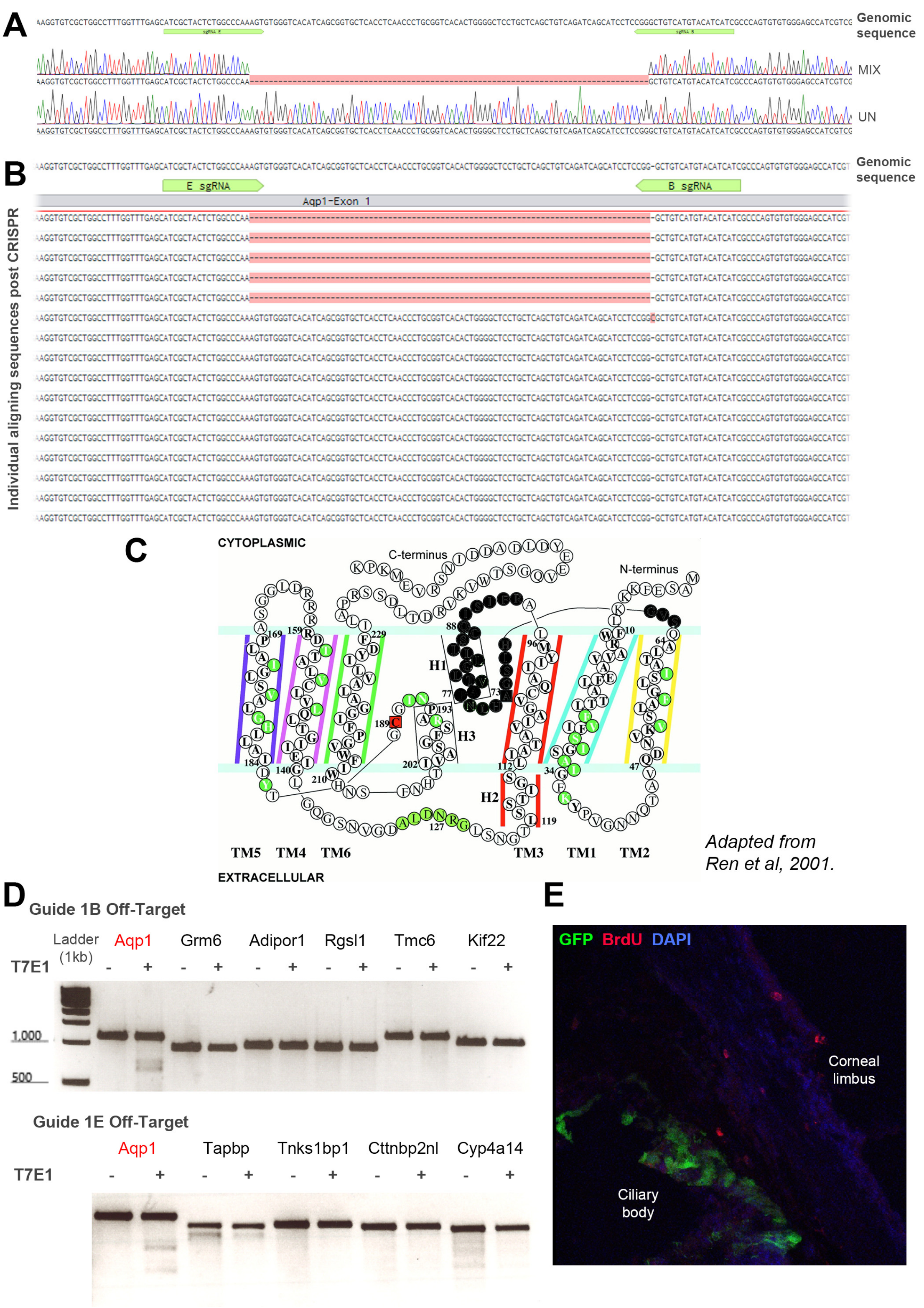

Figure S2. Representative sequencing chromatograms (**A**) from ShH10-CMV-SaCas9-sgRNA B and E (MIX) and un-infected control (UN) B6-RPE07 cells after 72 hours. Graphical depiction of on-target genomic DNA alterations (**B**) of further 16 accurately sequenced clones, where the predominant gene edit observed (31.2%) was complete excision of the 98bp intervening region between the cleavage points of the two sgRNA guides. This excised DNA region corresponds to critical central amino acid residues

shaded in black on the polypeptide sequence (**C**) adapted from *Ren et al.*[1] T7 endonuclease 1 assay for the top five bioinformatics predicted off-target coding genes (**D**) for sgRNA B and E do not demonstrate genomic cleavage.[2] Tested 72 hours following plasmid transfection into B6-RPE07 cells. Each shown with and without addition of T7E1 enzyme, with *Aqp1* positive control. To exclude ShH10 viral infection causing ciliary body non-pigmented epithelium cell turnover, BrdU was administered 24 hours before cryosection and staining, but 3 weeks after intravitreal injection of ShH10-CMV-eGFP. Representative image (**E**) of n=3 eyes showing expected corneal limbal transient amplifying cells as positive control. There is minimal colocalization of cells in the GFP+ ciliary epithelium with BrdU.

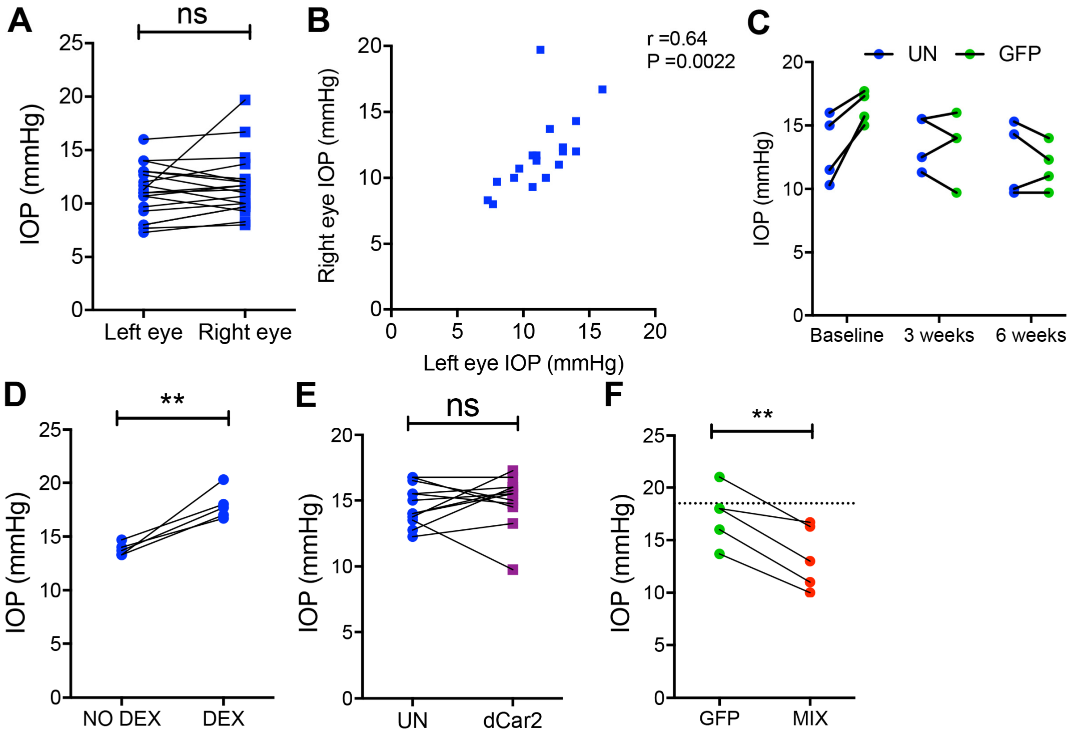

Figure S3. Additional *in vivo* data. Comparison of left and right eye intraocular pressures (IOP) prior to any intervention demonstrating no statistical difference by paired analysis (**A**, paired Student's t test, n = 19, p = 0.31) and good correlation (**B**, Pearson correlation, r = 0.64, p = 0.0022). This supports use of the contralateral eye as a control with lower inter- than intra-ocular variance (4.86 vs 5.44mmHg). IOP measurement of contralateral eyes injected with GFP vector demonstrates no statistically significant reduction from the vector across time (**C**, Repeated measures two-way ANOVA, n = 4, p = 0.52). Confirmation of the magnitude of IOP elevation in the corticosteroid (Dexamethasone) ocular hypertension model is shown as IOP after 3 weeks with contralateral eye control (**D**, paired Student's t test, n = 5, p = 0.0052). Delivery of catalytically inactive Cas9 and sgRNA targeting a different gene (*Car2*) does not reduce IOP (**E**, n = 12, paired Student's t test, n = 12, p = 0.62), implying potential immune response against intravitreal injection, AAV or Cas9 expression may not affect IOP. GFP control vector was injected contralaterally alongside the MIX vector in an independent experiment one week following microbead model induction. After three weeks, reduced IOP is still observed (**F**, paired Student's t test, n = 5, P = 0.005, dotted line is mean IOP pre-AAV injection). Mean ± SEM shown. ns = not statistically significant.

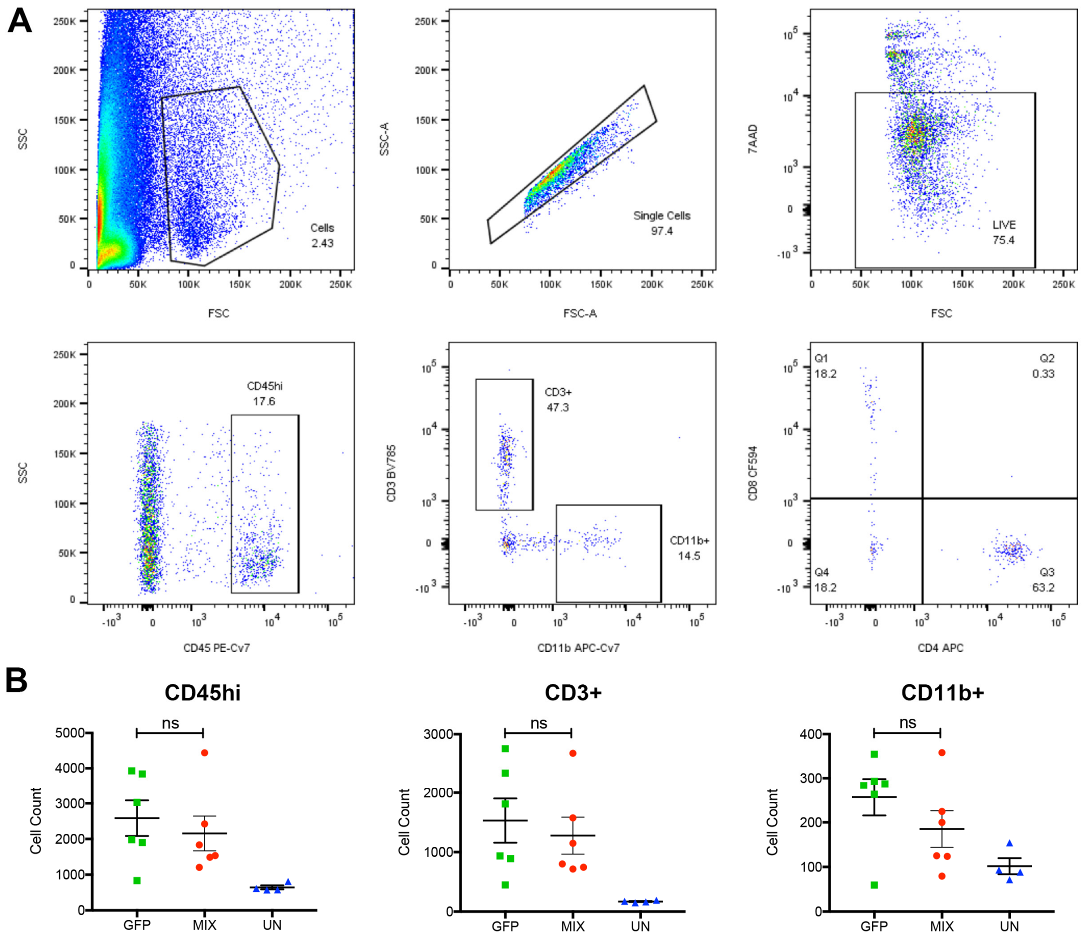

Figure S4. Ciliary body flow cytometry does not demonstrate differences in immune cell infiltration between the CRISPR-Cas9 and GFP control vectors. Whilst immune responses in the eye following intravitreal AAV injection can occur, we fail to identify differences between MIX and GFP that could confound the reduction in IOP observed. Eyes were injected with either ShH10-CMV-eGFP, un-injected (UN) or *Aqp1* targeting MIX. Three weeks later the ciliary body was extracted and analysed by flow cytometry for immune cell infiltration. **A**) Gating strategy on representative MIX eye and **B**) summary graph of absolute cell counts for total infiltrating leukocytes ($CD45^{Hi}$), lymphocytes (CD45+CD3+) or myeloid (CD45+CD11b+) populations. One-way ANOVA with Holm-Sidak's multiple comparison test between GFP and MIX, n=6 eyes per group. Mean ± SEM shown. ns = not statistically significant.

Supplemental Tables

Supplemental Table 1. CRISPR-SaCas9 sgRNA sequences

Target gene	sgRNA name	sgRNA sequence
Mouse *Aqp1 exon 1*	A	GATGTGACCCACACTTTGGGC
	B	GATGATGTACATGACAGCCCG
	C	TCTTCTGGAGGGCTGTGGTGG
	D	ACCAATGCTGATGAAGACGAA
	E	ATCGCTACTCTGGCCCAAAGT
	F	TAGGGAGGAGGTGATGCCCGA
Human *AQP1 exon 1*	H	CTGAGCATCGCCACGCTGGCG
	J	ACCGATGCTGATGAAGACAAA
	K	CCGCCGTCTGGTTGTTCCCCA

Supplemental Table 2. Primers for endpoint PCR and qPCR

Application and gene target	Forward Primer	Reverse Primer
T7 endonuclease 1 assay - Mouse *Aqp1* exon 1	CTAGAGTGCCAGCCTCTGCCCT	GGAGGAACTGCTGGCATGCACC
T7 endonuclease 1 assay - Human *AQP1* exon 1	CTGCATCCATCCAGAGGAGGTC	GACAGGCTGGGCATTTGGTTC
Endpoint PCR for SaCas9 DNA sequence	AGCAAGGCCCTGGAAGAGAAAT	TGGTGGGGATCTCTTTCTGCTG

Supplemental Table 3. T7 endonuclease 1 assay primers for predicted region of CRISPR-Cas9 off-target coding genes

sgRNA	Predicted off-target gene	Forward Primer	Reverse Primer
m*Aqp1* B	Tmc6	CTCGCACATGAGCACTGTCTTC	TGGCTCAGCAATTAAGAGGGCT
m*Aqp1* B	Kif22	GCACTTCCTTCTGCTGGCAAAA	CTACTGGCTGGAAGGAAGTGCT
m*Aqp1* B	Grm6	CTATAGGTAGCGCAGGAACCCC	TATGCACCCCTTAGGCACTCAC
m*Aqp1* B	Adipor1	CACCACTATGCTAGCTCCCTGT	GGGACAGTGAGCTGACATGTCA
m*Aqp1* B	Rgsl1	ATGGGACCTGATGTGTGTCCTG	GATGGTTTCTGCCTTGGCAGAC
m*Aqp1* E	Tapbp	ATTGTGCCCTTAGAGATCCGGG	CTCTGAGCTCCCACTTGACCTC
m*Aqp1* E	Tnks1bp1	TTCTTCCATCTGCCTCCCTGTG	TCACCACGTCCTCCTCAGTAGA
m*Aqp1* E	Cttnbp2nl	TAATGGCCACTGTGACCCAGAG	GGGTTTAACGCTGAGCTCTTGG
m*Aqp1* E	Cyp4a14	TCGCTTTAACTCTGACCAGCCA	TGGTGAGAGTGGGGTGTTGAAG

Supplemental Materials & Methods

CRISPR-Cas9 indel formation characterisation

B6-RPE07 at 70% confluency in a 24-well plate were transfected using Lipofectamine 3000 (Thermo Fisher Scientific, UK) with a 50:50 mix of pX601-AAV-CMV:NLS-SaCas9-NLS-3xHA-bGHpA;U6::BsaI-sgRNA plasmid containing sgRNA mAqp1 B or E. After 72 hours genomic DNA was isolated using DNeasy Blood and Tissue Kit (Qiagen, Germany) and the edited exon 1 *mAqp1* region amplified using PCR Master Mix (Thermo Fisher Scientific, UK) and primers 5'-CTAGAGTGCCAGCCTCTGCCCT-3' and 5'- GGAGGAACTGCTGGCATGCACC-3'. The resulting amplicon mix was ligated into pGEM-T Easy plasmid (Promega Corporation, WI, USA) per manufacturer's instructions and transformed into DH5α competent cells (Thermo Fisher Scientific, UK). Twenty individual colonies were isolated, plasmid DNA extracted and sent for Sanger sequencing (Eurofins Genomics, Germany) using primer 5'-TGTAAAACGACGGCCAGT-3'. Alignment of resulting sequences was performed using Benchling.

BrdU cell division assay

One-hundred microlitres of 10mg/ml Bromodeoxyuridine solution (BD Biosciences, CA, USA) was injected intraperitoneally into mice 24-hours prior to termination. Eyes were enucleated and cryo-sectioned at 14µm intervals as described previously. After washing with PBS, sections were incubated with 2N HCL at 37°C for 15 minutes then processed with primary and secondary antibodies as per manufacturer's instructions (eBioscience™ BrdU Kit for IHC/ICC Immunofluorescence eFluor™ 570, Thermo Fisher Scientific, UK). Sections were imaged by confocal laser scanning microscopy on a Leica SP5 (Leica Microsystems, Germany).

Flow cytometry of Ciliary Body

Eyes were dissected in 100µl PBS with ciliary body, iris and aqueous extracted following a limbal incision, lens removal and transfer to a 1.5 mL microcentrifuge tube. Tissue was mechanically dissociated rapping the tube across an 80-well rack ten times before transfer into a 96-well 60 µm cell strainer plate (Merck Millipore, UK) which was centrifuged at 1200 rpm for 5 minutes before the cell pellet was resuspended in 0.1% BSA in PBS buffer and transferred into a 96-well V-bottom plate. Cell pellets were incubated with purified rat anti-mouse CD16/32 Fc block (1:50, 553142, [2.4G2], BD Biosciences (BD)) for 10 minutes on ice before incubation with fluorochrome-conjugated monoclonal antibodies against mouse immune cell surface markers: CD45 [1:1500, 552848, [30-F11], BD), CD3 (1:40, 100355, [145-2C11], BioLegend), CD4 (1:100, 553051, [RM4-5], BD), CD8 (1:200, 126622, [YTS156.7.7], BioLegend) and CD11b (1:200, 557657, [M1/70], BD) at 4°C for 20 minutes. Following washing cells were resuspended in 200 µl 7-aminoactinomycin D (7AAD; Thermo Fisher) and acquired using a fixed and stable flow rate for 4 minutes on a 4-laser Fortessa X20 flow cytometer (BD Cytometry Systems, UK). Compensation was performed using OneComp eBeads (01-1111-41, ThermoFisher) and eight two-fold serial dilutions of a known concentration of AccuCheck Counting Beads (PCB100, ThermoFisher) were similarly acquired to construct a standard curve to calculate absolute cell numbers. Final analysis was performed using FlowJo software (Treestar, California, USA).

Supplemental References

1. Hsu P, Scott D, Weinstein J, Ran F, Konermann S, Agarwala V, *et al* (2013). DNA targeting specificity of RNA-guided Cas9 nucleases. *Nature Biotechnology* **31**:827-32.
